# phytochrome B Is Required for Light-Mediated Systemic Control of Stomatal Development

**DOI:** 10.1016/j.cub.2014.03.074

**Published:** 2014-06-02

**Authors:** Stuart A. Casson, Alistair M. Hetherington

**Affiliations:** 1 Department of Molecular Biology and Biotechnology, University of Sheffield, Firth Court, Western Bank, Sheffield S10 2TN, UK; 2 School of Biological Sciences, University of Bristol, Woodland Road, Bristol BS8 1UG, UK

## Abstract

Stomata are pores found on the surfaces of leaves, and they regulate gas exchange between the plant and the environment [1]. Stomatal development is highly plastic and is influenced by environmental signals [2]. Light stimulates stomatal development, and this response is mediated by plant photoreceptors [3–5], with the red-light photoreceptor phytochrome B (phyB) having a dominant role in white light [3]. Light also regulates stomatal development systemically, with the irradiance perceived by mature leaves modulating stomatal development in young leaves [6, 7]. Here, we show that phyB is required for this systemic response. Using a combination of tissue-specific expression and an inducible expression system in the loss-of-function *phyB-9* mutant [8], we show that phyB expression in the stomatal lineage, mesophyll, and phloem is sufficient to restore wild-type stomatal development. Induction of *PHYB* in mature leaves also rescues stomatal development in young untreated leaves, whereas *phyB* mutants are defective in the systemic regulation of stomatal development. Our data show that phyB acts systemically to regulate cell fate decisions in the leaf epidermis.

## Highlights

• phyB is required during the light-mediated systemic control of stomatal development
• Light-mediated stomatal development does not require phyB expression in the epidermis
• *PHYB* expression in mature *phyB*-9 leaves rescues stomatal development in young leaves
• *phyB-9* mutants are defective in light-mediated systemic stomatal development

## Teaser

Stomatal development is regulated by light, which requires the red-light photoreceptor phytochrome B. By using both tissue-specific and inducible phyB expression, Casson and Hetherington show that phyB acts both within the stomatal lineage and systemically to regulate stomatal development.

## Results

**Figure 1:**
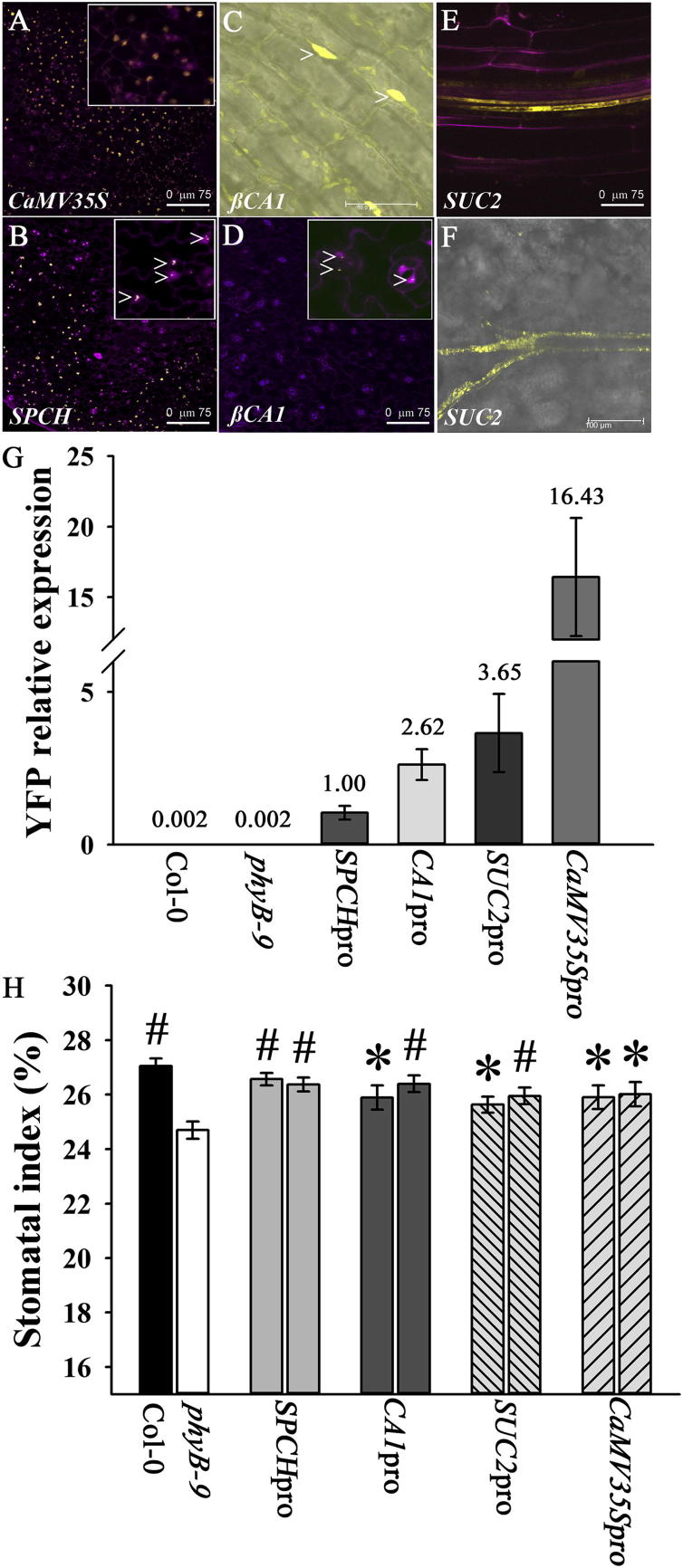
Tissue-Specific Expression of phyB Confocal microscope images of tissue-specific *PHYB::YFP* lines. (A and B) Overlaid images. YFP is shown in the yellow channel; tissue was counterstained with propidium iodide (magenta channel). (A) *CaMV35sproPHYB::YFP*; abaxial epidermis (the scale bar represents 75 μm). Inset shows epidermal cells expressing *CaMV35sproPHYB::YFP*. (B) *SPCHproPHYB::YFP*; abaxial epidermis 7 days postgermination (dpg) (the scale bar represents 75 μm). Inset shows stomatal lineage cells expressing *SPCHproPHYB::YFP* (arrowheads). (C) *βCA1proPHYB::YFP*; palisade cells 7 dpg; YFP channel only (the scale bar represents 50 μm). Arrowheads highlight YFP expression in palisade cells. YFP is shown in the yellow channel overlying the bright-field image. (D and E) Overlaid images. YFP is shown in the yellow channel; tissue was counterstained with propidium iodide (magenta channel). (D) *βCA1proPHYB::YFP*; abaxial epidermis 7 dpg (the scale bar represents 75 μm). Inset shows guard cells expressing *βCA1proPHYB::YFP* (arrowheads). (E) *Suc2proPHYB::YFP*; seedling root shows companion cell expression surrounding the vascular tissue (7 dpg; the scale bar represents 75 μm). (F) *Suc2proPHYB::YFP*; subepidermal tissue. YFP expression in companion cells surrounding vascular tissue (7 dpg; the scale bar represents 100 μm). YFP is shown in the yellow channel overlying the bright-field image. (G) Relative expression of *PHYB-YFP* as determined by qPCR in selected lines using YFP-specific primers. RNA was extracted from 2-week-old seedlings. Number above each column indicates expression calculated relative to that of *SPCHproPHYB::YFP*. Error bars indicate mean ± SEM from three biological replicates. (H) SI of mature leaves (L11–13) of tissue-specific *PHYB::YFP* lines grown at 250 μmol m^−2^ s^−1^. Mean values are shown for two independent transformed lines per construct with error bars indicating mean ± SEM. Symbols indicate significant difference in SI compared to *phyB-9*; ^∗^p < 0.05, #p < 0.005. See also Figure S1.

Plants regulate gas exchange in the short term by adjusting the aperture of the stomatal pore in response to biotic and abiotic signals [1]. In addition, plants regulate the number of stomata that develop on leaves [2]. This involves changes in epidermal cell fate that result in alterations to the stomatal index (SI; the ratio of the number of stomata in a given area divided by the total number of stomata and other epidermal cells in that area) and changes in stomatal density (SD). Genes involved in the basal signaling pathway underlying stomatal development include the basic-helix-loop-helix (bHLH) transcription factors *SPEECHLESS* (*SPCH*), *MUTE*, and *FAMA*, which control consecutive steps in the differentiation of mature guard cells, and various peptides such as STOMAGEN [9–12]. Increased expression of these factors during the early stages of leaf development leads to increased recruitment of epidermal cells into the stomatal lineage and changes in SI and SD [9–12]. Light regulates plant development [13], and increased photon irradiances, regulated by plant photoreceptors, result in increases in SI [3–5]. phyB has a dominant role in white light, with *phyB* mutants having reduced SI at higher photon irradiances [3, 4]. *phyB* is expressed widely throughout the life cycle of *Arabidopsis* and throughout the leaf, including in early stomatal lineage cells, guard cells, and pavement cells [5, 14]. Although this would suggest that phyB can act cell autonomously to regulate phyB-dependent responses, mesophyll-specific expression was found to be sufficient to suppress flowering, indicating that some phyB responses are regulated non-cell autonomously [15]. To investigate in which tissues and cells phyB is required to mediate stomatal development, we used tissue-specific promoters to drive the expression of a PHYB-YFP fusion protein (Figure S1A available online). They included a promoter that drives expression within the stomatal lineage, a promoter that directs expression within nonepidermal leaf tissues, and a constitutive promoter. Constructs were stably introduced into the *phyB-9* mutant (Col-0 background) by *Agrobacterium tumefaciens*-mediated transformation, and two independent transformed lines were analyzed per promoter construct and gave similar expression patterns. The spatial expression of the PHYB-YFP fusion protein was determined by confocal microscopy in the first pair of true leaves of 7-day-old soil-grown seedlings (Figures 1A–1F). For representative lines, the level of transgene expression was also determined by quantitative PCR (qPCR) using primers specific to *YFP* (Figure 1G). The phenotype of mature plants is shown in Figure S1B.

### PHYB-YFP Expression within the Stomatal Lineage Complements the *phyB*-Deficient Stomatal Development Phenotype

When grown in white light at a photon irradiance of 250 μmol m^−2^ s^−1^, mature leaves of the loss-of-function *phyB-9* mutant had a significantly reduced SI compared with the Col-0 control (Figure 1H). Under these conditions, SD was also significantly reduced (Col-0 SD: 228.3 mm^2^ ± SEM 4.7; *phyB-9* SD: 201.8 mm^2^ ± SEM 5.4; p = 0.0005). This reduced SI phenotype could be rescued when the PHYB-YFP fusion protein was expressed constitutively in the *phyB-9* background using the *CaMV35S* promoter, which included epidermal expression (Figures 1A and 1H). To determine whether the expression of PHYB within the stomatal lineage is required for ensuring that the appropriate SI is achieved, we expressed the PHYB-YFP fusion protein using the *SPCH* promoter (Figures 1B and S1A) that directs expression throughout the stomatal lineage [9]. The presence of the PHYB-YFP fusion protein (Figure 1B; *SPCH* promoter) resulted in rescue of the *phyB-9* stomatal mutant phenotype (Figure 1H). Expression of PHYB-YFP using the *βCA1* promoter also rescued the *phyB*-9 stomatal mutant phenotype. This promoter directed PHYB-YFP expression most strongly in mesophyll cells; however, weak expression was also detected in guard cells [16] (Figures 1C, 1D, and 1H). Analysis of promoter *PHYB::YFP* transgenic lines indicated that SI was not directly associated with transgene level; for example, *SPCH*pro*PHYB::YFP* and *CaMV35S*pro*PHYB::YFP* lines have almost identical SIs, and, yet, transgene expression is 16-fold higher in the *CaMV35*Spro*PHYB::YFP* line (Figure 1G). Furthermore, although tissue-specific expression of *PHYB::YFP* led to changes in leaf size (Figure S1B), these changes did not correlate with the ability to rescue the SI defect of *phyB-9* plants. This agrees with recent work showing that differentiation in the stomatal lineage is independent of pavement cell expansion, which makes the major contribution to overall leaf size [17].

### PHYB-YFP Expression in the Phloem Rescues the *phyB* Mutant Stomatal Development Phenotype

phyB can act in a non-cell-autonomous manner to regulate flowering in *Arabidopsis* [15]. To determine whether this is the case for stomatal development, we expressed PHYB-YFP in nonepidermal cells using the *SUC2* promoter (phloem companion cells; Figures 1E and 1F). Confocal microscopy confirmed that PHYB-YFP expression was not observed in the epidermis (including guard cells) and was restricted to the phloem (Figure 1F), whereas leaf impressions revealed that expression of PHYB-YFP in the phloem is sufficient to rescue stomatal development in the *phyB-9* mutant (Figure 1H).

### phyB Acts Systemically to Control Stomatal Development

**Figure 2:**
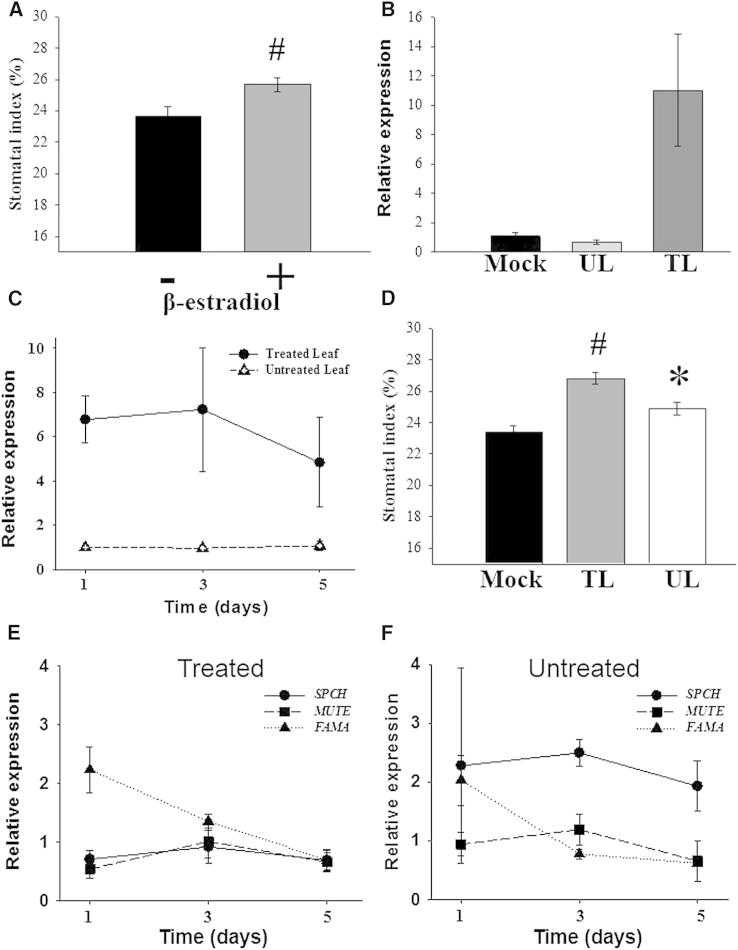
Inducible Expression of phyB (A) SI of i-*PHYB* plants grown at 250 μmol m^−2^ s^−1^. Whole plants were sprayed daily with either a mock treatment (−) or 5 μM β-estradiol (+). Mean values are shown with error bars indicating mean ± SEM. Symbols indicate significant difference in SI compared to the control (−); #p < 0.005. (B) *PHYB* expression determined by qPCR in i-*PHYB* leaves. 5 μM β-estradiol was applied to individual leaves with a paintbrush, and *PHYB* expression was monitored 48 hr later on both treated (TL) and untreated (UL) leaves on the same plant. Expression is relative to mock-treated i-*PHYB* plants (Mock). Error bars indicate mean ± SEM. (C) *PHYB* expression determined by qPCR in both TL and UL of i-*PHYB* plants. 5 μM β-estradiol was applied daily to individual leaves with a paintbrush, and *PHYB* expression was monitored in both the TL (L1–12; •) and young UL (L13–15; Δ) from the same plant over a time course (1, 3, and 5 days from first treatment). Expression is relative to the equivalent leaves (L1–12 for treated and 13–15 for untreated) from mock-treated i-*PHYB* plants. Error bars indicate mean ± SEM from three biological replicates. (D) The SI of i-*PHYB* plants grown at 250 μmol m^−2^ s^−1^. Initial mature leaves (TL) of individual plants were treated by painting leaves daily with 5 μM β-estradiol, whereas young leaves (UL) were untreated (Figure S2D). Mean values are shown with error bars indicating mean ± SEM. Symbols indicate significant difference in SI compared with the control (Mock); ^∗^p < 0.05, #p < 0.005. (E and F) Quantitative gene expression analysis of *SPCH*, *MUTE*, and *FAMA* in i-*PHYB* leaves. As in (C), 5 μM β-estradiol was applied with a paintbrush to individual leaves daily, and gene expression was monitored in both the TL (L1–12; E) and young UL (L13–15; F) from the same plant over a time course (1, 3, and 5 days from first treatment). Expression is relative to the equivalent leaves from mock-treated i-*PHYB* plants. Error bars indicate mean ± SEM from three biological replicates. See also Figure S2.

Although the analysis of tissue-specific *PHYB::YFP* lines reveals the spatial requirement for phyB during stomatal development, it does not distinguish between a local requirement within a developing leaf and a systemic role in existing mature leaves. To address this issue, an inducible *PHYB* system was constructed in the *phyB-9* mutant background. A line (i-*PHYB*) was chosen that was phenotypically indistinguishable from the *phyB-9* mutant (indicating that if there was any leaky *PHYB* expression in this line, it was insufficient to complement the mutant phenotype; Figures S2A and 2A, untreated). To test for functionality, we sprayed all leaves of i-*PHYB* plants with 5 μM β-estradiol. This resulted in increased *PHYB* expression and rescue of both the gross *phyB-9* mutant phenotype and stomatal development (Figures S2A, S2B, and 2A). β-estradiol treatment alone did not significantly alter stomatal development of Col-0 and *phyB-9* plants (Figure S2C). The β-estradiol-inducible expression system shows tight spatial regulation of transgene induction [18]. *PHYB* expression was monitored by qPCR, and this confirmed that the induction of *phyB* expression was restricted to leaves treated with β-estradiol (and that this treatment did not result in induction or spread of *PHYB* expression in nontreated tissue; Figures 2B and 2C).

To determine whether phyB can act systemically to influence stomatal development, we treated leaves (L) 1–12 daily by applying β-estradiol to them. This treatment began once leaves were greater than 5 mm in length (7 days postgermination for L1) and continued for another 3 weeks as new leaves developed. Young leaves were left untreated (Figure S2D). SI was measured in both the β-estradiol-treated leaves (L10–12) and the younger untreated leaves (L13–15). Both the treated leaves (TL) and the young untreated leaves (UL) showed significant increases in SI compared with the mock-treated control (Mock) (Figure 2D).

The expression of several genes known to be involved in the control of stomatal developmental is reduced in *phyB* mutants [4, 5]. To determine whether the changes in SI in i-*PHYB* plants are due to changes in the expression of one or more positive regulators of stomatal development, we analyzed the expression of the transcription factors *SPCH*, *MUTE*, and *FAMA* [9–11] by qPCR following β-estradiol treatment in both TL and young UL from the same plants. Compared with equivalent mock-treated plants, *FAMA* expression was elevated in TL, whereas *SPCH* expression increased in new leaves (Figures 2E and 2F).

### *phyB* Mutants Are Defective in Systemic Regulation of Light-Induced Stomatal Development

**Figure 3:**
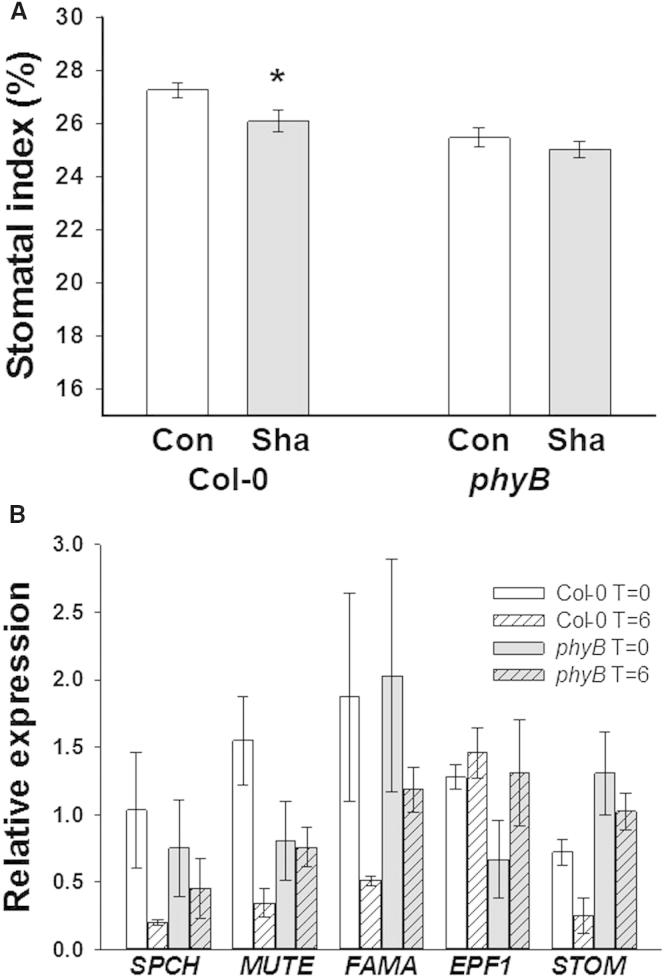
phyB Is Required for the Systemic Regulation of Stomatal Development (A) *phyB-9* mutants do not respond to shading. Plants were germinated at a photon irradiance of 250 μmol m^−2^ s^−1^, and on emergence of L14, developed leaves were shaded with neutral density filters to a photon irradiance of 50 μmol m^−2^ s^−1^, with L14 exposed to 250 μmol m^−2^ s^−1^ (Sha). Control SI of the equivalent leaf from plants grown 250 μmol m^−2^ s^−1^ are shown for comparison (Con). Mean values are shown with error bars indicating mean ± SEM. Symbols indicate significant difference in SI compared to the control (250); ^∗^p < 0.05. (B) Quantitative gene expression analysis of stomatal developmental genes in Col-0 and *phyB-9* mutants following shading. Plants were grown as in (A), and RNA was extracted from L14 at t = 0 and t = 6 hr postshading of mature leaves. Expression is relative to L14 from unshaded plants (Col-0 or *phyB-9*, respectively) at the equivalent time points. Error bars indicate mean ± SEM from three biological replicates (Student’s t test p values for t = 0 compared with t = 6; *SPCH*: Col-0 = 0.12, *phyB-9* = 0.51; *MUTE*: Col-0 = 0.02, *phyB-9* = 0.89; *FAMA*: Col-0 = 0.15, *phyB-9* = 0.39; *STOM*: Col-0 = 0.09, *phyB-9* = 0.39). See also Figure S3.

Mature leaves can signal to developing leaves to regulate stomatal development in response to light and CO_2_ [6, 7]. Both the tissue-specific expression of PHYB-YFP and inducible expression of *PHYB* reported here support a role for phyB in the systemic regulation of stomatal development. We therefore investigated whether the *phyB-9* mutant shows defects in this process. Wild-type (WT) and *phyB-9* were grown at high light (250 μmol m^−2^ s^−1^) until the initiation of L14 primordia. Maturing L1–13 were then shaded using a neutral density filter, (resulting in a photon irradiance of 50 μmol m^−2^ s^−^), while L14 was exposed to and continued to develop at high light (250 μmol m^−2^ s^−1^; Figure S3A). Consistent with similar experiments [6, 7], L14 of Col-0 plants had a reduced SI compared with equivalent high-light-grown leaves (Figure 3A). In contrast, there was no significant reduction of SI of L14 in *phyB-9* mutants, indicating that *phyB-9* mutants are defective in this systemic signaling pathway. Gene expression in L14 was compared with the equivalent leaf from plants grown at high light (250 μmol m^−2^ s^−1^). At 6 hr after the shading of mature leaves, the expression of the positive regulators of stomatal development *SPCH*, *MUTE*, *FAMA*, and *STOMAGEN* (*STOM*) [9–12] was reduced in the exposed L14 of Col-0 plants. *phyB-9* mutants did not show equivalent reductions in the expression of these key regulators (Figure 3B).

## Discussion

Previous work showed that light controls stomatal development [3, 5–7] and that this involves phyB [3, 4]. It was also known that this is a systemic response [6, 7]. Here, we demonstrate that phyB acts both within the stomatal lineage and in nonepidermal tissue to regulate cell fate changes during stomatal development. phyB also acts in the systemic pathway to modulate stomatal development in young leaves in response to light signals perceived by mature leaves.

As expression throughout the stomatal lineage rescues the reduced SI phenotype of the *phyB-9* mutant, phyB may therefore act locally at the earliest stages of stomatal development to modulate epidermal cell fate decisions. Changes in stomatal number correlate with leaf transpiration rate, suggesting that perturbations in guard cell function can affect stomatal development [19]. In this context, it is important to note that phyB is required for red-light-induced stomatal opening [20]. It is possible, therefore, that the reduced SI in *phyB-9* mutants is the result of reductions in stomatal aperture at high light, a phenotype that is likely complemented in the *SPCH*pro*PHYB::YFP*, *βCA1*pro*PHYB::YFP*, and *CaMV35S*pro*PHYB::YFP* lines, which all direct *PHYB::YFP* expression in guard cells (as well as in other cell types). However, such a possibility is not consistent with the ability of phloem-expressed PHYB-YFP to complement the *phyB-9* phenotype (Figure 1H). Instead, our data suggest that there is phyB-mediated intertissue signaling that regulates cell fate changes in the epidermis.

To determine whether phyB is part of a systemic signaling pathway, we engineered the *phyB-9* mutant to express WT *PHYB* inducibly (i-*PHYB* plants). The induction of *PHYB* led to an initial increase in *FAMA* expression in TL (Figure 2E), consistent with previous reports showing that phyB regulates *FAMA* expression [4]. In the epidermis of *phyB-9* leaves grown at high light (250 μmol m^−2^ s^−1^), some amplifying divisions are seen to terminate prematurely without differentiating into satellite stomata (Figures S3B and S3C). This would be consistent with a defect in the latter stages of stomatal development, which are regulated by FAMA. Given the experimental timescale (6 hr posttreatment), phyB-mediated signaling may directly control an upstream regulator of *FAMA* expression.

Treatment of only the mature leaves of i-*PHYB* plants with β-estradiol resulted in significant increases in SI of the young UL (Figure 2D) and increased *SPCH* expression in young UL (Figure 2F). Because SPCH is required for initiation of stomatal development [9], these data indicate that changes in stomatal development in young leaves are likely to occur de novo in response to a phyB-mediated signal from mature leaves. When mature leaves of WT plants are shaded, young leaves develop with reduced SI compared with high-light-grown plants (Figure 3A) [6, 7]. This reduction in SI correlated with downregulated expression of the positive regulators of stomatal development *SPCH*, *MUTE*, *FAMA*, and *STOMAGEN*. *EPF1*, like *STOMAGEN*, is a member of the *EPIDERMAL PATTERNING FACTOR-LIKE* (*EPFL*) family and regulates one-cell spacing [21]. The expression of *EPF1* was not significantly affected. This result might reflect differences in the function of individual genes influenced by the systemic signaling pathway or may be due to the sampling time series chosen for this experiment. In contrast, *phyB-9* mutant young leaves are indistinguishable from high-light-grown controls and do not show downregulation in expression of these positive regulators of stomatal development (Figure 3B). Taken together with the i-*PHYB* data, our results demonstrate that phyB is required for light-mediated systemic regulation of stomatal development, and this systemic mechanism is likely dependent on changes in the expression of stomatal development genes in young leaves.

A question remains as to the nature of the systemic signal generated in response to light perception by phyB. It has been reported that phyB acts upstream of the MAPK signaling cascade headed by the MAPKKK YDA [5, 22] that targets the SPCH protein and potentially MUTE and FAMA [23, 24]. Although the data presented here show that transcriptional changes in *SPCH*, *MUTE*, and *FAMA* occur in young leaves during systemic signaling, our data do not exclude additional posttranscriptional regulatory signaling mechanisms. Integration of signals through the MAPK module is emerging as a common mechanism for regulating stomatal development with YODA and SPCH targeted by brassinosteroid [25, 26]. It will therefore be interesting to investigate whether phyB operates through this module, and if it does, whether it forms part of the systemic system investigated here. Interestingly, PHYTOCHROME-INTERACTING FACTOR 4 (PIF4) acts with phyB to mediate light-regulated changes in stomatal development [3]. PIF4 interacts with the brassinosteroid-activated transcription factor BZR1 [27]; however, the integration point between brassinosteroid signaling and stomatal development is both upstream and independent of BZR1 [25, 26]. PIF4 also regulates auxin biosynthesis [28], and auxin has recently been shown to be required for correct stomatal development [29]. Although PIF4 regulation of auxin biosynthesis presents a potential mechanism by which light may mediate changes in stomatal development, this requires further work.

## Experimental Procedures

### *PHYB* Induction

For inducible *PHYB* experiments, *phyB-9* plants containing pMDC150-35S and pMDC221-*PHYB* were treated with 5 μM β-estradiol (Sigma-Aldrich) dissolved in water containing 0.01% (v/v) Silwet L-77 (Lehle Seeds). For Figures 2A and S2A–S2C, whole plants were treated by spraying leaves from 7 days postgermination for 3 weeks. For Figure 2B, 5 μM β-estradiol was applied to mature leaves (TL; L1–12) of 3-week-old plants with a fine paintbrush, and *PHYB* expression was monitored 48 hr later on both TL and UL on the same plant. For Figures 2C, 2E, and 2F, 5 μM β-estradiol was applied daily for 5 days to mature leaves (TL; L1–12) of 3-week-old plants with a fine paintbrush, with gene expression analysis performed on both treated (L1–12) and untreated (L13–15) leaves at 1, 3, and 5 days after treatment. For Figure 2D, 5 μM β-estradiol was applied to mature leaves (TL; L1–12) once a leaf was >5 mm in length (7 days postgermination for L1). Treatment was continued daily as new leaves developed for 3 weeks. Mock treatments were performed with 0.01% Silwet L-77. Treatments were applied 30 min predawn (Figure S2A).

### Shading Experiments

Plants were germinated at a photon irradiance of 250 μmol m^−2^ s^−1^. Once L14 primordia were visible (<2 mm), existing leaves (greater than 5 mm; designated mature) were shaded to 50 μmol m^−2^ s^−1^ using neutral density filters (Lee Filters), with L14 exposed to 250 μmol m^−2^ s^−1^ (Figure S3A). Equivalent leaves (L14) from unshaded plants were used as controls.

## Supplemental Information

Document S1. Supplemental Experimental Procedures and Figures S1–S3

Document S2. Article plus Supplemental Information
