# Supplementary material for: phytochrome B Is Required for Light-Mediated Systemic Control of Stomatal Development
Source: Curr Biol. 2014 Jun 2;24(11):1216–21. doi: 10.1016/j.cub.2014.03.074 (PMC4046225; doi:10.1016/j.cub.2014.03.074)
Supplement: Document S1. Supplemental Experimental Procedures and Figures S1–S3 [file mmc1.pdf]

Current Biology, Volume *24*

Supplemental Information

phytochrome B Is Required for Light-Mediated Systemic Control of Stomatal Development

Stuart A. Casson and Alistair M. Hetherington

Figure S1, related to Figure 1.

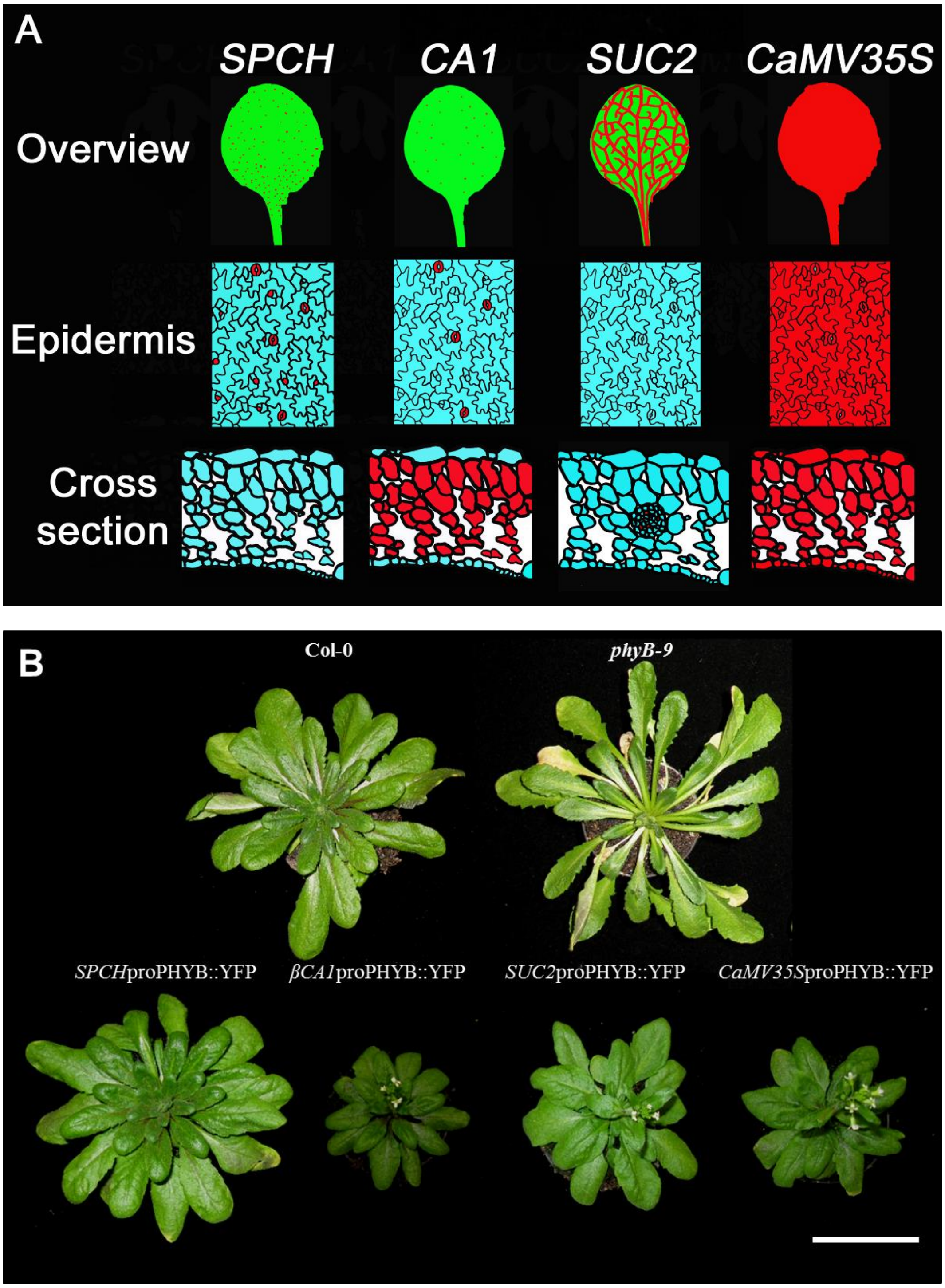

Figure S1, related to Figure 1. Tissue specific expression of *PHYB::YFP*.

A) Schematic detailing the expression patterns of the different tissue-specific promoters in leaf tissue as described in this study. Red indicates cells expressing PHYB::YFP

B) Rosette phenotype of mature plants (scale bar = 5cm).

Figure S2, related to Figure 2

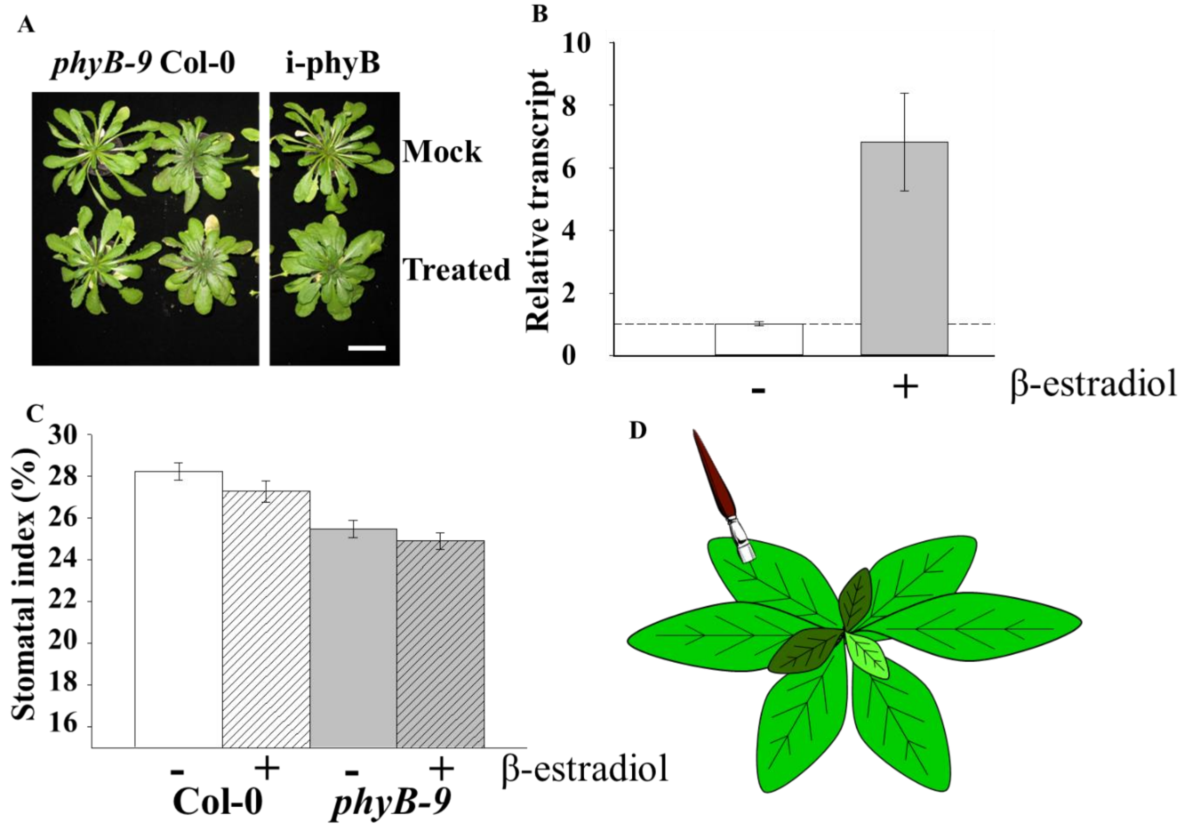

Figure S2, related to Figure 2. Inducible phytochrome B.

A) Phenotype of mature plants grown as in Figure 2A (scale bar = 5cm).

B) Quantitative RT-PCR showing induction of *PHYB* expression following treatment with 5µM β-estradiol. 2 week old seedlings were sprayed with 5µM β-estradiol and RNA extracted from mock treated (-) and treated (+) plants 2 days post treatment. Expression is calculated relative to the untreated control and error bars show mean +/- SEM.

C) The SI of Col-0 and *phyB-9* plants mock treated (-) or treated with 5µM β-estradiol (+). Plants were grown at 250µmol m^{-2} s^{-1} and sprayed daily. Mean values are shown with error bars indicating mean +/- SEM. No significant difference was observed within genotypes.

D) For determining the systemic role of *PHYB*, 5µM β-estradiol was applied to mature leaves (1-12) with a fine paint brush but not to young leaves (dark and light green).

Figure S3, related to Figure 3

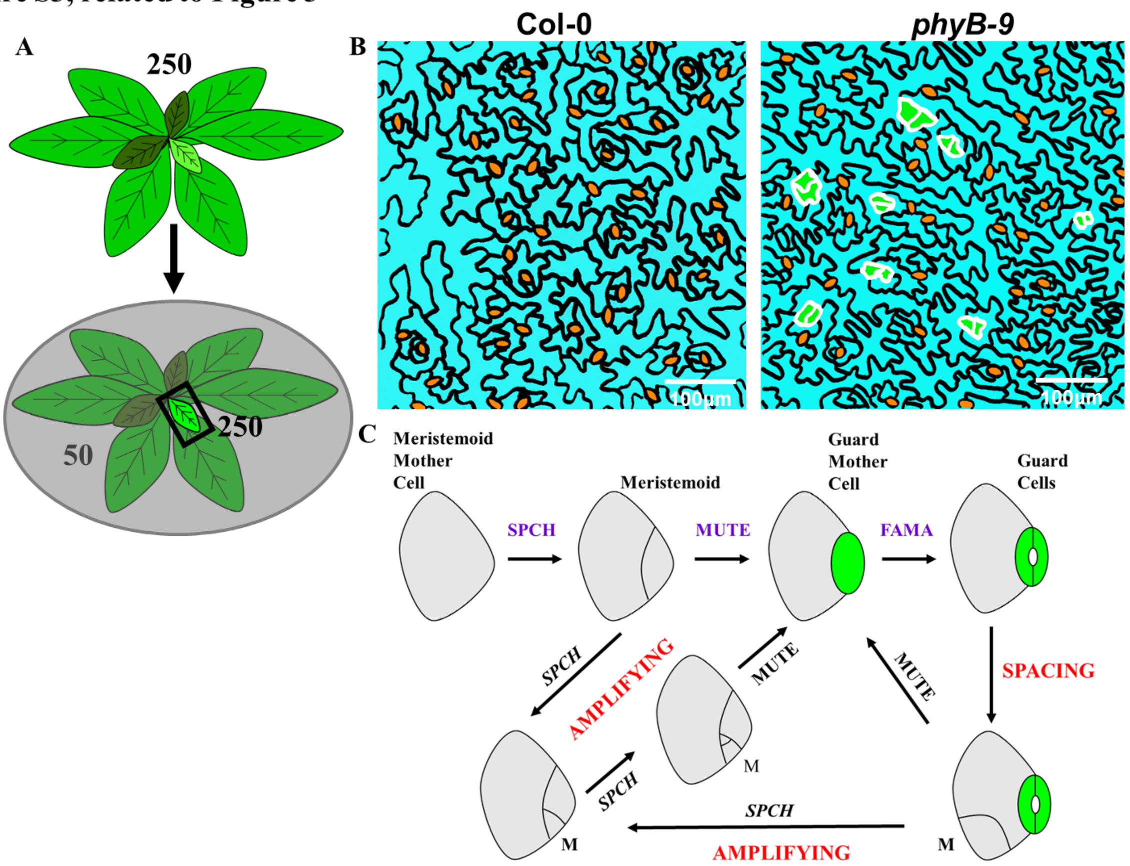

Figure S3, related to Figure 3. Scheme for shading and stomatal development

A) Plants were germinated at 250 µmol m^{-2} s^{-1} until leaf 14 primordia were visible. The existing leaves (greater than 5 mm; designated mature) were shaded to 50 µmol m^{-2} s^{-1} using neutral density filters whilst leaf 14 (light green) remained exposed to 250 µmol m^{-2} s^{-1}.

B) *phyB-9* mutants display aborted amplifying divisions. Epidermal tracings from mature leaves. Aborted divisions in the stomatal lineage are highlighted in white with cells filled in green; mature stomata are shown in orange (scale bar = 100µm).

C) A schematic indicating cell divisions within the stomatal lineage and the relevant roles of SPCH, MUTE and FAMA. Entry into the stomatal lineage occurs via an asymmetric division to generate a meristemoid cell, which requires SPCH. Differentiation into a Guard Mother Cell requires MUTE, whilst Guard Cell differentiation requires FAMA. Spacing and amplifying divisions result in further cells entering the stomatal lineage and generate satellite stomata.

Supplemental Experimental Procedures

Plant Material and Growth Conditions

Arabidopsis plants were all in the Col-0 ecotype. *phyB-9* is a strong mutant allele caused by a G-A transition leading to a premature STOP codon at amino acid 397 [8]. Phytochrome nomenclature is as detailed in [S1]. Therefore, phyB refers to the holoprotein, *PHYB* the wild-type gene, *phyB* the mutant and PHYB the apoprotein. Plants were grown in growth chambers (Snijder Microclima 1000E, Snijder Scientific, The Netherlands) from seed in 3:1 mix of compost-horticultural silver sand in short days (10 hr photoperiod, 70% RH, 22 °C) at a photon irradiance of 250 µmol m^{-2} s^{-1} unless otherwise stated.

Measurement of Stomatal Indices and Density

Impressions of the abaxial surface of mature rosette leaves, principal growth stage 5.10 [S2], were made with dental resin (President Jet Light Body, Coltene/Whaledent, Burgess Hill, UK). This was performed for leaves at comparable developmental stages. In the case of the tissue specific promoter analysis, two independent lines were analysed per construct. Clear nail varnish was applied to the set impression after removal from the leaf, and the varnish impressions were viewed on a Zeiss Axiovert 200M inverted microscope and imaged with Volocity software (Improvision Ltd, Coventry, UK). Stomatal and epidermal cell counts were taken from three areas per leaf with three leaves per plant from four separate plants and each experiment was performed in triplicate. For the density data, the mean was calculated from the total number of stomata or epidermal cells. The stomatal index was calculated for each area individually, and the mean was then calculated from these data. Stomatal index was calculated with the following formula: S.I. = [number of stomata/(number of other epidermal cells + number of stomata)] X 100. For statistical analysis, an unpaired t test was performed on the data following arcsine transformation, which was performed because stomatal index is a proportion and not a direct measurement.

Plasmid construction

PromoterPHYB::*YFP*

A full-length *PHYB* cDNA minus the TAG STOP codon was amplified from cDNA using phyBfor and phyBrev primers. This was then digested with *KpnI*, a site for which was added to the phyBrev primer. YFP was amplified from vector pGKGWY [S3] and was also digested with *KpnI*, a site for which was added to the YFPfor primer. The digested fragments were ligated together and then digested with *ApaI* (phyBfor primer) and *SbfI* (YFPrev primer). This product was then ligated into *ApaI-SbfI* digested pGKGWY to generate pGKGWY-*PHYB*. Promoters were amplified from genomic DNA (Col-0), digested with the relevant restriction endonculeases, and ligated into the *XbaI-ApaI* sites (*SPCH* 2060 bp; *SUC2* 2128 bp; *CaMV35S* 555 bp) or the *ApaI* site (*βCA1*1713 bp; At3g01500) of pGKGWY-*PHYB*.

Inducible PHYB

The Inducible *PHYB* line (i-*PHYB*) was generated using the β-estradiol inducible two-component vector system designed by Brand et al. [18]. The *PHYB* cDNA was amplified from Col-0 cDNA using the primers 221-phyBfor/rev and inserted into *AscI-PacI* digested pMDC221. A *CaMV35S* promoter was amplified from the binary vector pBI121 [S4] using the primers 150-35Sfor/rev and inserted into *AscI-PacI* digested pMDC150. Constructs were introduced into *phyB-9* mutants by the floral-dip method [S5] using the *Agrobacterium tumefaciens* C58C1 [S6]. Primary transformants were selected on growth medium supplemented with either 50 mg l^{-1} kanamycin (pMDC150-35S) or 20 mg l^{-1} hygromycin B (pMDC221-*PHYB*). Primary transformants displaying a *phyB-9* mutant phenotype (indicating a lack of non-specific transgene induction) were crossed and then selfed to generate a homozygous line containing both pMDC150-35S and pMDC221-phyB. Confirmation of

homozygosity for the *phyB-9* mutation was determined using the *phyB-9* specific primers *phyB-9*for/rev.

Details of primer sequences for vector construction can be found in the supplemental experimental procedures.

Confocal Microscopy

Confocal images were taken with a Leica TCS SP5 confocal microscope after counterstaining tissues with 10 µg ml^{-1} propidium iodide.

Gene expression analysis

For quantitative RT-PCR analysis (qPCR), RNA was extracted from 2 week old seedlings or leaves using the Qiagen RNeasy plant RNA extraction kit. 2µg of total RNA was treated with DNase according to the method of Sanyal et al. [S7], and then was reverse transcribed with Maxima reverse transcriptase (Fermentas). Transcript abundance of target genes was assayed using Maxima SYBR Green/ROX qPCR Master mix (Fermentas). The *ACTIN2* and *UBC21* genes were used as controls, as transcript levels remained constant under all treatments and relative expression levels were calculated using the ΔΔCt method [S8]. Expression was calculated relative to that of equivalent leaves from mock treated plants. Three biological repeats and three technical repeats were performed for each sample and used to calculate s.e.m. values. Reaction conditions were (1 x 95°C - 10 mins; 40 x 95°C - 15s/57°C – 20s/72°C – 30s). Details of primer sequences for gene expression analysis can be found in these supplemental experimental procedures.

Primers for quantitative RT-PCR

Primer	Forward (5'-3')<br>Reverse (5'-3')
ACTIN2	TCAGATGCCCAGAAGTGTGTT<br>CCGTACAGATCCTTCCTGATAT
UBC21	GAATGCTTGGAGTCCTGCTTG<br>CTCAGGATGAGCCATCAATGC
YFP	CTTCAAGGACGACGGCAACTAC<br>TTCAGCTCGATGCGGTTCAC
phyB	CCGTGACATTCCCGAAGAGAT<br>ATACTCAGCAGAAACTCAGCCA
SPCH	AACGGTGTCGCATAAGATCC<br>CAAGAGCCAAATCTTCAAGAGC
MUTE	AACGTCGAAAGACCCTAAACCG<br>TTAGCATGAGGGGAGTTACAGC
FAMA	GCTGCTAGGGTTTGACGCCATGA<br>GGAGTAGAGGACGGTTTGTTCC
EPF1	CCAACATCCTCCCATCCAAGTC<br>TGAGCAATCTGGCAACCTAGACC
STOMAGEN	GTTCAAGCCTCAAGACCTCG<br>CCTTCGACTGGAACTTGCTC

Primers for vector construction and genotyping

Primer	Forward (5'-3')<br>Reverse (5'-3')
phyB	TTAGGGCCCCAAACGGCATGGTTTCCGGAGTCG<br>TTAGGTACCATATGGCATCATCAGCATCATGTC
YFP	TTAGGTACCGTGGTGCTCGAGATGGTGAGC<br>CAGCGCATGCCTGCAGGTCGAC
SPCH	TTTCTAGAGTAACGGGCAACAGTGTTATAG<br>TTGGGCCCCGTGATTAGAGATATATCCT
SUC2	TTTCTAGAAAAATCTGGTTTCATATTAATTTCA<br>TTGGGCCCATTTGACAAACCAAGAAAGTAAGA
βCA1	TTGGGCCCGATCTGTAGGGTTGAGCAACTAGC<br>TTGGGCCCGCGAAGAGAAGCGGAGATCAGAG
CaMV35S	TTTCTAGAGAATTCCCATGGAGTCAAAAATTC<br>TTGGGCCCGGTACCGTGTCCTCTCCAAATGA
150-35S	GAGGCGCGCCTCAAATAGAGGACCTAACAG<br>AGTTAATTAATGTCCTCTCCAAATGA
221-phyB	TTGGCGCGCCCAAACGGCATGGTTTCCGGAGTCG<br>GGTTAATTAACCAACTGAAGTGTGACTAATATGGC
Wt-phyB	CTATGTGCTTGGTTGGTTCTAC<br>GATGGCAAACAACCAAACCC
phyB-9	GAAGCTCGATGAGGCTTTGA<br>CCATGATACTGGGACTCTGTG

Supplemental References

S1. Srivastava, L.M. (2002). Plant Growth and Development: Hormones and Environment (Elsevier Inc.).

S2. Boyes, D.C., Zayed, A.M., Ascenzi, R., McCaskill, A.J., Hoffman, N.E., Davis, K.R., and Gorlach, J. (2001). Growth stage-based phenotypic analysis of Arabidopsis: A model for high throughput functional genomics in plants. Plant Cell *13*, 1499–1510.

S3. Zhong, S., Lin, Z., Fray, R., Grierson, D. (2008). Improved plant transformation vectors for fluorescent protein tagging. Transgenic Res *17,* 985-989.

S4. Jefferson, R.A., Kavangh. T,A., Bevan, M.W. (1987). GUS fusions: β-glucuronidase as a sensitive and versatile gene fusion marker in higher plants. EMBO J *6*, 3901–3907.

S5. Clough, S.J., Bent, A.F. (1998). Floral dip: a simplified method for Agrobacterium-mediated transformation of Arabidopsis thaliana. Plant J *6*, 735–743.

S6. Dale, P.J., Marks, M.S., Brown, M.M., Woolston, C.J., Gunn, H.V., Mullineaux, P.M., Lewis, D.M., Kemp, J.M., Chen, D.F., Gilmour, D.M., Flavell, R.B. (1989). Agroinfection of wheat-inoculation of in vitro grown seedlings and embryos. Plant Sci *63*, 237–245.

S7. Sanyal, A., O'Driscoll, S.W., Bolander, M.A., Sarkar, G. (1997). An effective method of completely removing contaminating genomic DNA from an RNA sample to be used for PCR. Mol Biotechnol *8,* 135–137.

S8. Schmittgen, T.D., Livak, K.J. (2008). Analyzing real-time PCR data by the comparative C(T) method. Nat Protoc. *3*, 1101-1108.
