# Supplementary material for: phytochrome B Is Required for Light-Mediated Systemic Control of Stomatal Development
Source: Curr Biol. 2014 Jun 2;24(11):1216–21. doi: 10.1016/j.cub.2014.03.074 (PMC4046225; doi:10.1016/j.cub.2014.03.074)
Supplement: Document S2. Article plus Supplemental Information [file mmc2.pdf]

Current Biology 24, 1216–1221, June 2, 2014 ©2014 The Authors http://dx.doi.org/10.1016/j.cub.2014.03.074

Report

phytochrome B Is Required for Light-Mediated Systemic Control of Stomatal Development

Stuart A. Casson[1,*] and Alistair M. Hetherington[2]
[1]Department of Molecular Biology and Biotechnology, University of Sheffield, Firth Court, Western Bank, Sheffield S10 2TN, UK
[2]School of Biological Sciences, University of Bristol, Woodland Road, Bristol BS8 1UG, UK

Summary

Stomata are pores found on the surfaces of leaves, and they regulate gas exchange between the plant and the environment [1]. Stomatal development is highly plastic and is influenced by environmental signals [2]. Light stimulates stomatal development, and this response is mediated by plant photoreceptors [3–5], with the red-light photoreceptor phytochrome B (phyB) having a dominant role in white light [3]. Light also regulates stomatal development systemically, with the irradiance perceived by mature leaves modulating stomatal development in young leaves [6, 7]. Here, we show that phyB is required for this systemic response. Using a combination of tissue-specific expression and an inducible expression system in the loss-of-function *phyB-9* mutant [8], we show that phyB expression in the stomatal lineage, mesophyll, and phloem is sufficient to restore wild-type stomatal development. Induction of *PHYB* in mature leaves also rescues stomatal development in young untreated leaves, whereas *phyB* mutants are defective in the systemic regulation of stomatal development. Our data show that phyB acts systemically to regulate cell fate decisions in the leaf epidermis.

Results

Plants regulate gas exchange in the short term by adjusting the aperture of the stomatal pore in response to biotic and abiotic signals [1]. In addition, plants regulate the number of stomata that develop on leaves [2]. This involves changes in epidermal cell fate that result in alterations to the stomatal index (SI; the ratio of the number of stomata in a given area divided by the total number of stomata and other epidermal cells in that area) and changes in stomatal density (SD). Genes involved in the basal signaling pathway underlying stomatal development include the basic-helix-loop-helix (bHLH) transcription factors *SPEECHLESS* (*SPCH*), *MUTE*, and *FAMA*, which control consecutive steps in the differentiation of mature guard cells, and various peptides such as STOMAGEN [9–12]. Increased expression of these factors during the early stages of leaf development leads to increased recruitment of epidermal cells into the stomatal lineage and changes in SI and SD [9–12]. Light regulates plant development [13], and increased photon irradiances, regulated by plant photoreceptors, result in increases in SI [3–5]. phyB has a dominant role in white light, with *phyB* mutants having reduced SI at higher photon irradiances [3, 4]. *phyB* is expressed widely throughout the life cycle of *Arabidopsis* and throughout the leaf, including in early stomatal lineage cells, guard cells, and pavement cells [5, 14]. Although this would suggest that phyB can act cell autonomously to regulate phyB-dependent responses, mesophyll-specific expression was found to be sufficient to suppress flowering, indicating that some phyB responses are regulated non-cell autonomously [15]. To investigate in which tissues and cells phyB is required to mediate stomatal development, we used tissue-specific promoters to drive the expression of a PHYB-YFP fusion protein (Figure S1A available online). They included a promoter that drives expression within the stomatal lineage, a promoter that directs expression within nonepidermal leaf tissues, and a constitutive promoter. Constructs were stably introduced into the *phyB-9* mutant (Col-0 background) by *Agrobacterium tumefaciens*-mediated transformation, and two independent transformed lines were analyzed per promoter construct and gave similar expression patterns. The spatial expression of the PHYB-YFP fusion protein was determined by confocal microscopy in the first pair of true leaves of 7-day-old soil-grown seedlings (Figures 1A–1F). For representative lines, the level of transgene expression was also determined by quantitative PCR (qPCR) using primers specific to *YFP* (Figure 1G). The phenotype of mature plants is shown in Figure S1B.

PHYB-YFP Expression within the Stomatal Lineage Complements the *phyB*-Deficient Stomatal Development Phenotype

When grown in white light at a photon irradiance of 250 μmol m^{-2} s^{-1}, mature leaves of the loss-of-function *phyB-9* mutant had a significantly reduced SI compared with the Col-0 control (Figure 1H). Under these conditions, SD was also significantly reduced (Col-0 SD: 228.3 mm^2 ± SEM 4.7; *phyB-9* SD: 201.8 mm^2 ± SEM 5.4; p = 0.0005). This reduced SI phenotype could be rescued when the PHYB-YFP fusion protein was expressed constitutively in the *phyB-9* background using the *CaMV35S* promoter, which included epidermal expression (Figures 1A and 1H). To determine whether the expression of PHYB within the stomatal lineage is required for ensuring that the appropriate SI is achieved, we expressed the PHYB-YFP fusion protein using the *SPCH* promoter (Figures 1B and S1A) that directs expression throughout the stomatal lineage [9]. The presence of the PHYB-YFP fusion protein (Figure 1B; *SPCH* promoter) resulted in rescue of the *phyB-9* stomatal mutant phenotype (Figure 1H). Expression of PHYB-YFP using the β*CA1* promoter also rescued the *phyB*-9 stomatal mutant phenotype. This promoter directed PHYB-YFP expression most strongly in mesophyll cells; however, weak expression was also detected in guard cells [16] (Figures 1C, 1D, and 1H). Analysis of promoter *PHYB::YFP* transgenic lines indicated that SI was not directly associated with transgene level; for example, *SPCH*pro*PHYB::YFP* and *CaMV35S*pro*PHYB::YFP* lines have almost identical SIs, and, yet, transgene expression is 16-fold higher in the *CaMV35S*pro*PHYB::YFP* line (Figure 1G). Furthermore, although tissue-specific expression of *PHYB::YFP* led to changes in leaf size (Figure S1B), these

*Correspondence: s.casson@sheffield.ac.uk
This is an open access article under the CC BY license (http://creativecommons.org/licenses/by/3.0/).

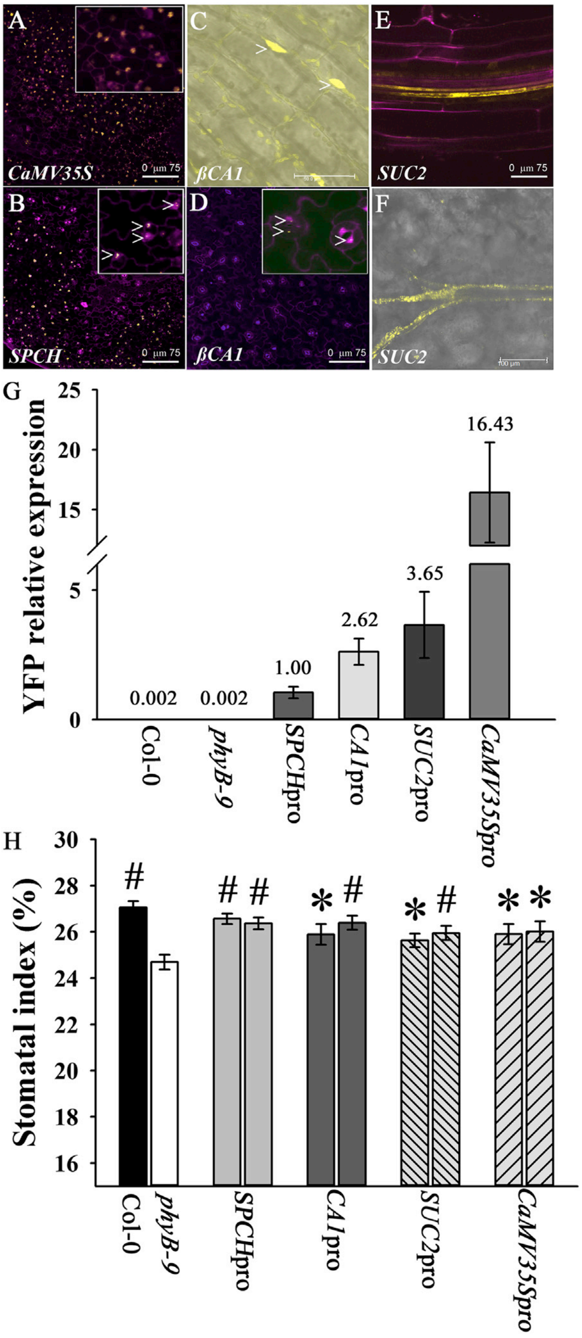

Figure 1. Tissue-Specific Expression of phyB

Confocal microscope images of tissue-specific *PHYB::YFP* lines.

(A and B) Overlaid images. YFP is shown in the yellow channel; tissue was counterstained with propidium iodide (magenta channel).

(A) *CaMV35sproPHYB::YFP*; abaxial epidermis (the scale bar represents 75 μm). Inset shows epidermal cells expressing *CaMV35sproPHYB::YFP*.

(B) *SPCHproPHYB::YFP*; abaxial epidermis 7 days postgermination (dpg) (the scale bar represents 75 μm). Inset shows stomatal lineage cells expressing *SPCHproPHYB::YFP* (arrowheads).

(C) *βCA1proPHYB::YFP*; palisade cells 7 dpg; YFP channel only (the scale bar represents 50 μm). Arrowheads highlight YFP expression in palisade cells. YFP is shown in the yellow channel overlying the bright-field image.

(D and E) Overlaid images. YFP is shown in the yellow channel; tissue was counterstained with propidium iodide (magenta channel).

(D) *βCA1proPHYB::YFP*; abaxial epidermis 7 dpg (the scale bar represents 75 μm). Inset shows guard cells expressing *βCA1proPHYB::YFP* (arrowheads).

(E) *Suc2proPHYB::YFP*; seedling root shows companion cell expression surrounding the vascular tissue (7 dpg; the scale bar represents 75 μm).

(F) *Suc2proPHYB::YFP*; subepidermal tissue. YFP expression in companion cells surrounding vascular tissue (7 dpg; the scale bar represents 100 μm). YFP is shown in the yellow channel overlying the bright-field image.

(G) Relative expression of *PHYB-YFP* as determined by qPCR in selected lines using YFP-specific primers. RNA was extracted from 2-week-old seedlings. Number above each column indicates expression calculated relative to that of *SPCHproPHYB::YFP*. Error bars indicate mean ± SEM from three biological replicates.

(H) SI of mature leaves (L11–13) of tissue-specific *PHYB::YFP* lines grown at 250 μmol m^{-2} s^{-1}. Mean values are shown for two independent transformed lines per construct with error bars indicating mean ± SEM. Symbols indicate significant difference in SI compared to *phyB-9*; *p < 0.05, #p < 0.005.

See also Figure S1.

changes did not correlate with the ability to rescue the SI defect of *phyB-9* plants. This agrees with recent work showing that differentiation in the stomatal lineage is independent of pavement cell expansion, which makes the major contribution to overall leaf size [17].

PHYB-YFP Expression in the Phloem Rescues the *phyB* Mutant Stomatal Development Phenotype

phyB can act in a non-cell-autonomous manner to regulate flowering in *Arabidopsis* [15]. To determine whether this is the case for stomatal development, we expressed PHYB-YFP in nonepidermal cells using the *SUC2* promoter (phloem companion cells; Figures 1E and 1F). Confocal microscopy confirmed that PHYB-YFP expression was not observed in the epidermis (including guard cells) and was restricted to the phloem (Figure 1F), whereas leaf impressions revealed that expression of PHYB-YFP in the phloem is sufficient to rescue stomatal development in the *phyB-9* mutant (Figure 1H).

phyB Acts Systemically to Control Stomatal Development

Although the analysis of tissue-specific *PHYB::YFP* lines reveals the spatial requirement for phyB during stomatal development, it does not distinguish between a local requirement within a developing leaf and a systemic role in existing mature leaves. To address this issue, an inducible *PHYB* system was constructed in the *phyB-9* mutant background. A line (i-*PHYB*) was chosen that was phenotypically indistinguishable from the *phyB-9* mutant (indicating that if there was any leaky *PHYB* expression in this line, it was insufficient to complement the mutant phenotype; Figures S2A and 2A, untreated). To test for functionality, we sprayed all leaves of i-*PHYB* plants with 5 μM β-estradiol. This resulted in increased *PHYB* expression and rescue of both the gross *phyB-9* mutant phenotype and stomatal development (Figures S2A, S2B, and 2A). β-estradiol treatment alone did not significantly alter stomatal development of Col-0 and *phyB-9* plants (Figure S2C). The β-estradiol-inducible expression system shows tight spatial regulation of transgene induction [18]. *PHYB* expression was monitored by qPCR, and this confirmed that the induction of *phyB* expression was restricted to leaves treated with β-estradiol (and that this treatment did not result in induction or spread of *PHYB* expression in nontreated tissue; Figures 2B and 2C).

To determine whether phyB can act systemically to influence stomatal development, we treated leaves (L) 1–12 daily by applying β-estradiol to them. This treatment began once leaves were greater than 5 mm in length (7 days postgermination for L1) and continued for another 3 weeks as new leaves

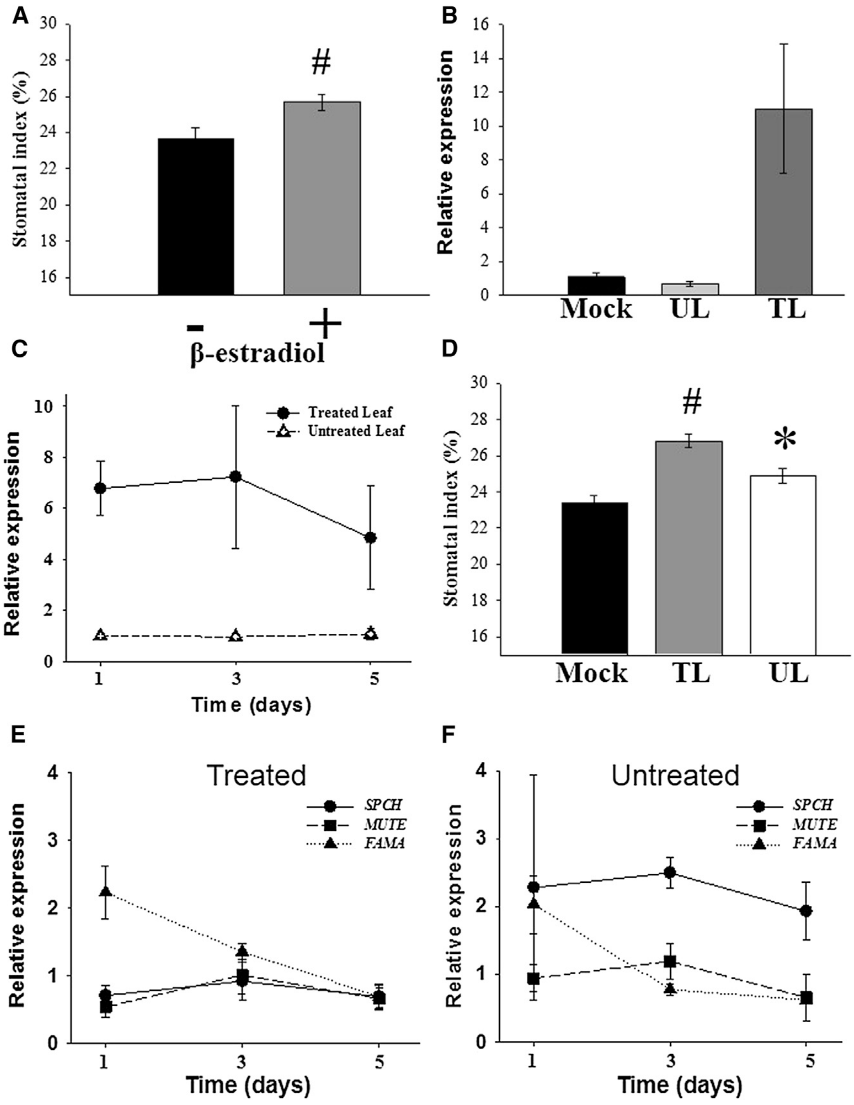

Figure 2. Inducible Expression of phyB

(A) SI of i-*PHYB* plants grown at 250 μmol m^{-2} s^{-1}. Whole plants were sprayed daily with either a mock treatment (−) or 5 μM β-estradiol (+). Mean values are shown with error bars indicating mean ± SEM. Symbols indicate significant difference in SI compared to the control (−); #$p < 0.005$.

(B) *PHYB* expression determined by qPCR in i-*PHYB* leaves. 5 μM β-estradiol was applied to individual leaves with a paintbrush, and *PHYB* expression was monitored 48 hr later on both treated (TL) and untreated (UL) leaves on the same plant. Expression is relative to mock-treated i-*PHYB* plants (Mock). Error bars indicate mean ± SEM.

(C) *PHYB* expression determined by qPCR in both TL and UL of i-*PHYB* plants. 5 μM β-estradiol was applied daily to individual leaves with a paintbrush, and *PHYB* expression was monitored in both the TL (L1–12; ●) and young UL (L13–15; Δ) from the same plant over a time course (1, 3, and 5 days from first treatment). Expression is relative to the equivalent leaves (L1–12 for treated and 13–15 for untreated) from mock-treated i-*PHYB* plants. Error bars indicate mean ± SEM from three biological replicates.

(D) The SI of i-*PHYB* plants grown at 250 μmol m^{-2} s^{-1}. Initial mature leaves (TL) of individual plants were treated by painting leaves daily with 5 μM β-estradiol, whereas young leaves (UL) were untreated (Figure S2D). Mean values are shown with error bars indicating mean ± SEM. Symbols indicate significant difference in SI compared with the control (Mock); *$p < 0.05$, #$p < 0.005$.

(E and F) Quantitative gene expression analysis of *SPCH*, *MUTE*, and *FAMA* in i-*PHYB* leaves. As in (C), 5 μM β-estradiol was applied with a paintbrush to individual leaves daily, and gene expression was monitored in both the TL (L1–12; E) and young UL (L13–15; F) from the same plant over a time course (1, 3, and 5 days from first treatment). Expression is relative to the equivalent leaves from mock-treated i-*PHYB* plants. Error bars indicate mean ± SEM from three biological replicates.

See also Figure S2.

developed. Young leaves were left untreated (Figure S2D). SI was measured in both the β-estradiol-treated leaves (L10–12) and the younger untreated leaves (L13–15). Both the treated leaves (TL) and the young untreated leaves (UL) showed significant increases in SI compared with the mock-treated control (Mock) (Figure 2D).

The expression of several genes known to be involved in the control of stomatal developmental is reduced in *phyB* mutants [4, 5]. To determine whether the changes in SI in i-*PHYB* plants are due to changes in the expression of one or more positive regulators of stomatal development, we analyzed the expression of the transcription factors *SPCH*, *MUTE*, and *FAMA* [9–11] by qPCR following β-estradiol treatment in both TL and young UL from the same plants. Compared with equivalent mock-treated plants, *FAMA* expression was elevated in TL, whereas *SPCH* expression increased in new leaves (Figures 2E and 2F).

phyB Mutants Are Defective in Systemic Regulation of Light-Induced Stomatal Development

Mature leaves can signal to developing leaves to regulate stomatal development in response to light and CO_2 [6, 7]. Both the tissue-specific expression of PHYB-YFP and inducible expression of *PHYB* reported here support a role for phyB in the systemic regulation of stomatal development. We therefore investigated whether the *phyB-9* mutant shows defects in this process. Wild-type (WT) and *phyB-9* were grown at high light (250 μmol m^{-2} s^{-1}) until the initiation of L14 primordia. Maturing L1–13 were then shaded using a neutral density filter, (resulting in a photon irradiance of 50 μmol m^{-2} s^{-}), while L14 was exposed to and continued to develop at high light (250 μmol m^{-2} s^{-1}; Figure S3A). Consistent with similar experiments [6, 7], L14 of Col-0 plants had a reduced SI compared with equivalent high-light-grown leaves (Figure 3A). In contrast, there was no significant reduction of SI of L14 in *phyB-9* mutants, indicating that *phyB-9* mutants are defective in this systemic signaling pathway. Gene expression in L14 was compared with the equivalent leaf from plants grown at high light (250 μmol m^{-2} s^{-1}). At 6 hr after the shading of mature leaves, the expression of the positive regulators of stomatal development *SPCH*, *MUTE*, *FAMA*, and *STOMAGEN* (*STOM*) [9–12] was reduced in the exposed L14 of Col-0 plants. *phyB-9* mutants did not show equivalent reductions in the expression of these key regulators (Figure 3B).

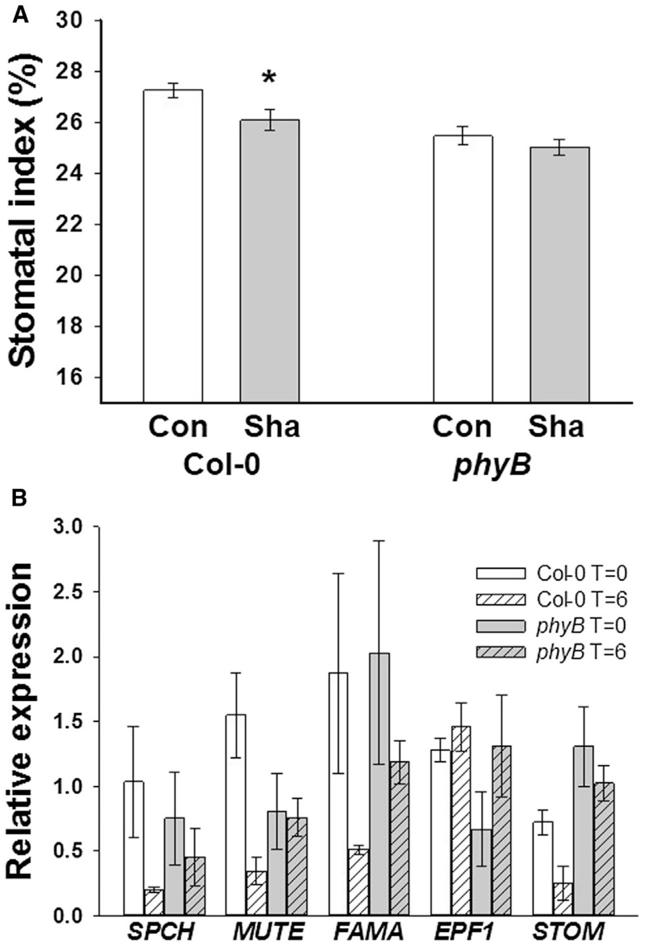

Figure 3. phyB Is Required for the Systemic Regulation of Stomatal Development

(A) *phyB-9* mutants do not respond to shading. Plants were germinated at a photon irradiance of 250 μmol m^{-2} s^{-1}, and on emergence of L14, developed leaves were shaded with neutral density filters to a photon irradiance of 50 μmol m^{-2} s^{-1}, with L14 exposed to 250 μmol m^{-2} s^{-1} (Sha). Control SI of the equivalent leaf from plants grown 250 μmol m^{-2} s^{-1} are shown for comparison (Con). Mean values are shown with error bars indicating mean ± SEM. Symbols indicate significant difference in SI compared to the control (250); *$p < 0.05$.

(B) Quantitative gene expression analysis of stomatal developmental genes in Col-0 and *phyB-9* mutants following shading. Plants were grown as in (A), and RNA was extracted from L14 at t = 0 and t = 6 hr postshading of mature leaves. Expression is relative to L14 from unshaded plants (Col-0 or *phyB-9*, respectively) at the equivalent time points. Error bars indicate mean ± SEM from three biological replicates (Student's t test p values for t = 0 compared with t = 6; *SPCH*: Col-0 = 0.12, *phyB-9* = 0.51; *MUTE*: Col-0 = 0.02, *phyB-9* = 0.89; *FAMA*: Col-0 = 0.15, *phyB-9* = 0.39; *STOM*: Col-0 = 0.09, *phyB-9* = 0.39).

See also Figure S3.

Discussion

Previous work showed that light controls stomatal development [3, 5–7] and that this involves phyB [3, 4]. It was also known that this is a systemic response [6, 7]. Here, we demonstrate that phyB acts both within the stomatal lineage and in nonepidermal tissue to regulate cell fate changes during stomatal development. phyB also acts in the systemic pathway to modulate stomatal development in young leaves in response to light signals perceived by mature leaves.

As expression throughout the stomatal lineage rescues the reduced SI phenotype of the *phyB-9* mutant, phyB may therefore act locally at the earliest stages of stomatal development to modulate epidermal cell fate decisions. Changes in stomatal number correlate with leaf transpiration rate, suggesting that perturbations in guard cell function can affect stomatal development [19]. In this context, it is important to note that phyB is required for red-light-induced stomatal opening [20]. It is possible, therefore, that the reduced SI in *phyB-9* mutants is the result of reductions in stomatal aperture at high light, a phenotype that is likely complemented in the *SPCH*pro*PHYB::YFP*, *βCA1*pro*PHYB::YFP*, and *CaMV35S*pro*PHYB::YFP* lines, which all direct *PHYB::YFP* expression in guard cells (as well as in other cell types). However, such a possibility is not consistent with the ability of phloem-expressed PHYB-YFP to complement the *phyB-9* phenotype (Figure 1H). Instead, our data suggest that there is phyB-mediated intertissue signaling that regulates cell fate changes in the epidermis.

To determine whether phyB is part of a systemic signaling pathway, we engineered the *phyB-9* mutant to express WT *PHYB* inducibly (i-*PHYB* plants). The induction of *PHYB* led to an initial increase in *FAMA* expression in TL (Figure 2E), consistent with previous reports showing that phyB regulates *FAMA* expression [4]. In the epidermis of *phyB-9* leaves grown at high light (250 μmol m^{-2} s^{-1}), some amplifying divisions are seen to terminate prematurely without differentiating into satellite stomata (Figures S3B and S3C). This would be consistent with a defect in the latter stages of stomatal development, which are regulated by FAMA. Given the experimental timescale (6 hr posttreatment), phyB-mediated signaling may directly control an upstream regulator of *FAMA* expression.

Treatment of only the mature leaves of i-*PHYB* plants with β-estradiol resulted in significant increases in SI of the young UL (Figure 2D) and increased *SPCH* expression in young UL (Figure 2F). Because SPCH is required for initiation of stomatal development [9], these data indicate that changes in stomatal development in young leaves are likely to occur de novo in response to a phyB-mediated signal from mature leaves. When mature leaves of WT plants are shaded, young leaves develop with reduced SI compared with high-light-grown plants (Figure 3A) [6, 7]. This reduction in SI correlated with downregulated expression of the positive regulators of stomatal development *SPCH*, *MUTE*, *FAMA*, and *STOMAGEN*. *EPF1*, like *STOMAGEN*, is a member of the *EPIDERMAL PATTERNING FACTOR-LIKE* (*EPFL*) family and regulates one-cell spacing [21]. The expression of *EPF1* was not significantly affected. This result might reflect differences in the function of individual genes influenced by the systemic signaling pathway or may be due to the sampling time series chosen for this experiment. In contrast, *phyB-9* mutant young leaves are indistinguishable from high-light-grown controls and do not show downregulation in expression of these positive regulators of stomatal development (Figure 3B). Taken together with the i-*PHYB* data, our results demonstrate that phyB is required for light-mediated systemic regulation of stomatal development, and this systemic mechanism is likely dependent on changes in the expression of stomatal development genes in young leaves.

A question remains as to the nature of the systemic signal generated in response to light perception by phyB. It has been reported that phyB acts upstream of the MAPK signaling

cascade headed by the MAPKKK YDA [5, 22] that targets the SPCH protein and potentially MUTE and FAMA [23, 24]. Although the data presented here show that transcriptional changes in *SPCH*, *MUTE*, and *FAMA* occur in young leaves during systemic signaling, our data do not exclude additional posttranscriptional regulatory signaling mechanisms. Integration of signals through the MAPK module is emerging as a common mechanism for regulating stomatal development with YODA and SPCH targeted by brassinosteroid [25, 26]. It will therefore be interesting to investigate whether phyB operates through this module, and if it does, whether it forms part of the systemic system investigated here. Interestingly, PHYTOCHROME-INTERACTING FACTOR 4 (PIF4) acts with phyB to mediate light-regulated changes in stomatal development [3]. PIF4 interacts with the brassinosteroid-activated transcription factor BZR1 [27]; however, the integration point between brassinosteroid signaling and stomatal development is both upstream and independent of BZR1 [25, 26]. PIF4 also regulates auxin biosynthesis [28], and auxin has recently been shown to be required for correct stomatal development [29]. Although PIF4 regulation of auxin biosynthesis presents a potential mechanism by which light may mediate changes in stomatal development, this requires further work.

Experimental Procedures

PHYB Induction

For inducible *PHYB* experiments, *phyB-9* plants containing pMDC150-35S and pMDC221-*PHYB* were treated with 5 μM β-estradiol (Sigma-Aldrich) dissolved in water containing 0.01% (v/v) Silwet L-77 (Lehle Seeds). For Figures 2A and S2A–S2C, whole plants were treated by spraying leaves from 7 days postgermination for 3 weeks. For Figure 2B, 5 μM β-estradiol was applied to mature leaves (TL; L1–12) of 3-week-old plants with a fine paintbrush, and *PHYB* expression was monitored 48 hr later on both TL and UL on the same plant. For Figures 2C, 2E, and 2F, 5 μM β-estradiol was applied daily for 5 days to mature leaves (TL; L1–12) of 3-week-old plants with a fine paintbrush, with gene expression analysis performed on both treated (L1–12) and untreated (L13–15) leaves at 1, 3, and 5 days after treatment. For Figure 2D, 5 μM β-estradiol was applied to mature leaves (TL; L1–12) once a leaf was >5 mm in length (7 days postgermination for L1). Treatment was continued daily as new leaves developed for 3 weeks. Mock treatments were performed with 0.01% Silwet L-77. Treatments were applied 30 min predawn (Figure S2A).

Shading Experiments

Plants were germinated at a photon irradiance of 250 μmol m^{-2} s^{-1}. Once L14 primordia were visible (<2 mm), existing leaves (greater than 5 mm; designated mature) were shaded to 50 μmol m^{-2} s^{-1} using neutral density filters (Lee Filters), with L14 exposed to 250 μmol m^{-2} s^{-1} (Figure S3A). Equivalent leaves (L14) from unshaded plants were used as controls.

Supplemental Information

Supplemental Information includes Supplemental Experimental Procedures and three figures and can be found with this article online at http://dx.doi.org/10.1016/j.cub.2014.03.074.

Acknowledgments

The authors would like to thank Kerry Franklin (University of Bristol) and Julie Gray (University of Sheffield) for comments on the manuscript. S.A.C. and A.M.H. acknowledge support from the BBSRC, grant BB/J002364. S.A.C. acknowledges support from the University of Sheffield.

Received: December 9, 2013
Revised: February 21, 2014
Accepted: March 28, 2014
Published: May 15, 2014

References

1. Hetherington, A.M., and Woodward, F.I. (2003). The role of stomata in sensing and driving environmental change. Nature *424*, 901–908.
2. Casson, S., and Gray, J.E. (2008). Influence of environmental factors on stomatal development. New Phytol. *178*, 9–23.
3. Casson, S.A., Franklin, K.A., Gray, J.E., Grierson, C.S., Whitelam, G.C., and Hetherington, A.M. (2009). Phytochrome B and PIF4 regulate stomatal development in response to light quantity. Curr. Biol. *19*, 229–234.
4. Boccalandro, H.E., Rugnone, M.L., Moreno, J.E., Ploschuk, E.L., Serna, L., Yanovsky, M.J., and Casal, J.J. (2009). Phytochrome B enhances photosynthesis at the expense of water-use efficiency in Arabidopsis. Plant Physiol. *150*, 1083–1092.
5. Kang, C.Y., Lian, H.L., Wang, F.F., Huang, J.R., and Yang, H.Q. (2009). Cryptochromes, phytochromes, and COP1 regulate light-controlled stomatal development in Arabidopsis. Plant Cell *21*, 2624–2641.
6. Lake, J.A., Quick, W.P., Beerling, D.J., and Woodward, F.I. (2001). Plant development. Signals from mature to new leaves. Nature *411*, 154.
7. Thomas, P.W., Woodward, F.I., and Quick, P.W. (2003). Systemic irradiance signalling in tobacco. New Phytol. *161*, 193–198.
8. Reed, J.W., Nagpal, P., Poole, D.S., Furuya, M., and Chory, J. (1993). Mutations in the gene for the red/far-red light receptor phytochrome B alter cell elongation and physiological responses throughout Arabidopsis development. Plant Cell *5*, 147–157.
9. MacAlister, C.A., Ohashi-Ito, K., and Bergmann, D.C. (2007). Transcription factor control of asymmetric cell divisions that establish the stomatal lineage. Nature *445*, 537–540.
10. Pillitteri, L.J., Sloan, D.B., Bogenschutz, N.L., and Torii, K.U. (2007). Termination of asymmetric cell division and differentiation of stomata. Nature *445*, 501–505.
11. Ohashi-Ito, K., and Bergmann, D.C. (2006). Arabidopsis FAMA controls the final proliferation/differentiation switch during stomatal development. Plant Cell *18*, 2493–2505.
12. Sugano, S.S., Shimada, T., Imai, Y., Okawa, K., Tamai, A., Mori, M., and Hara-Nishimura, I. (2010). Stomagen positively regulates stomatal density in Arabidopsis. Nature *463*, 241–244.
13. Chen, M., Chory, J., and Fankhauser, C. (2004). Light signal transduction in higher plants. Annu. Rev. Genet. *38*, 87–117.
14. Somers, D.E., and Quail, P.H. (1995). Temporal and spatial expression patterns of PHYA and PHYB genes in Arabidopsis. Plant J. *7*, 413–427.
15. Endo, M., Nakamura, S., Araki, T., Mochizuki, N., and Nagatani, A. (2005). Phytochrome B in the mesophyll delays flowering by suppressing FLOWERING LOCUS T expression in Arabidopsis vascular bundles. Plant Cell *17*, 1941–1952.
16. Hu, H., Boisson-Dernier, A., Israelsson-Nordström, M., Böhmer, M., Xue, S., Ries, A., Godoski, J., Kuhn, J.M., and Schroeder, J.I. (2010). Carbonic anhydrases are upstream regulators of CO2-controlled stomatal movements in guard cells. Nat. Cell Biol. *12*, 87–93, 1–18.
17. Andriankaja, M., Dhondt, S., De Bodt, S., Vanhaeren, H., Coppens, F., De Milde, L., Mühlenbock, P., Skirycz, A., Gonzalez, N., Beemster, G.T., and Inzé, D. (2012). Exit from proliferation during leaf development in Arabidopsis thaliana: a not-so-gradual process. Dev. Cell *22*, 64–78.
18. Brand, L., Hörler, M., Nüesch, E., Vassalli, S., Barrell, P., Yang, W., Jefferson, R.A., Grossniklaus, U., and Curtis, M.D. (2006). A versatile and reliable two-component system for tissue-specific gene induction in Arabidopsis. Plant Physiol. *141*, 1194–1204.
19. Lake, J.A., and Woodward, F.I. (2008). Response of stomatal numbers to CO2 and humidity: control by transpiration rate and abscisic acid. New Phytol. *179*, 397–404.
20. Wang, F.F., Lian, H.L., Kang, C.Y., and Yang, H.Q. (2010). Phytochrome B is involved in mediating red light-induced stomatal opening in Arabidopsis thaliana. Mol. Plant *3*, 246–259.
21. Hara, K., Kajita, R., Torii, K.U., Bergmann, D.C., and Kakimoto, T. (2007). The secretory peptide gene EPF1 enforces the stomatal one-cell-spacing rule. Genes Dev. *21*, 1720–1725.
22. Bergmann, D.C., Lukowitz, W., and Somerville, C.R. (2004). Stomatal development and pattern controlled by a MAPKK kinase. Science *304*, 1494–1497.
23. Lampard, G.R., Macalister, C.A., and Bergmann, D.C. (2008). Arabidopsis stomatal initiation is controlled by MAPK-mediated regulation of the bHLH SPEECHLESS. Science *322*, 1113–1116.
24. Lampard, G.R., Lukowitz, W., Ellis, B.E., and Bergmann, D.C. (2009). Novel and expanded roles for MAPK signaling in Arabidopsis stomatal

cell fate revealed by cell type-specific manipulations. Plant Cell *21*, 3506–3517.
25. Gudesblat, G.E., Schneider-Pizoń, J., Betti, C., Mayerhofer, J., Vanhoutte, I., van Dongen, W., Boeren, S., Zhiponova, M., de Vries, S., Jonak, C., and Russinova, E. (2012). SPEECHLESS integrates brassinosteroid and stomata signalling pathways. Nat. Cell Biol. *14*, 548–554.
26. Kim, T.W., Michniewicz, M., Bergmann, D.C., and Wang, Z.Y. (2012). Brassinosteroid regulates stomatal development by GSK3-mediated inhibition of a MAPK pathway. Nature *482*, 419–422.
27. Oh, E., Zhu, J.Y., and Wang, Z.Y. (2012). Interaction between BZR1 and PIF4 integrates brassinosteroid and environmental responses. Nat. Cell Biol. *14*, 802–809.
28. Franklin, K.A., Lee, S.H., Patel, D., Kumar, S.V., Spartz, A.K., Gu, C., Ye, S., Yu, P., Breen, G., Cohen, J.D., et al. (2011). Phytochrome-interacting factor 4 (PIF4) regulates auxin biosynthesis at high temperature. Proc. Natl. Acad. Sci. USA *108*, 20231–20235.
29. Le, J., Liu, X.G., Yang, K.Z., Chen, X.L., Zou, J.J., Wang, H.Z., Wang, M., Vanneste, S., Morita, M., Tasaka, M., et al. (2014). Auxin transport and activity regulate stomatal patterning and development. Nat Commun *5*, 3090.

Current Biology, Volume *24*

Supplemental Information

phytochrome B Is Required for Light-Mediated Systemic Control of Stomatal Development

Stuart A. Casson and Alistair M. Hetherington

Figure S1, related to Figure 1.

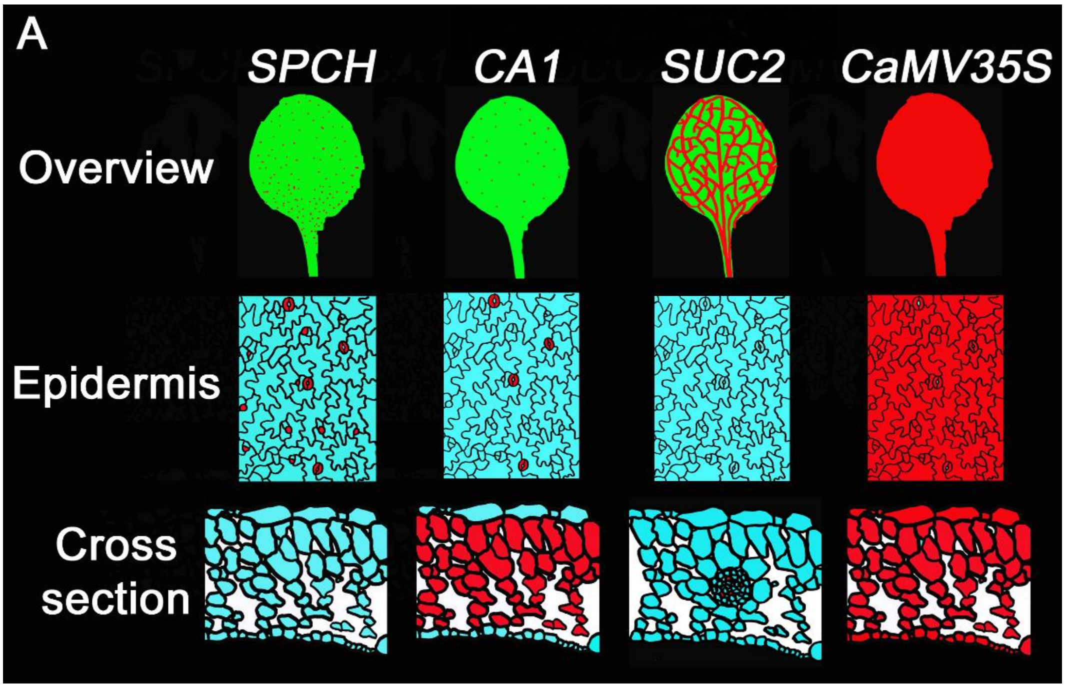

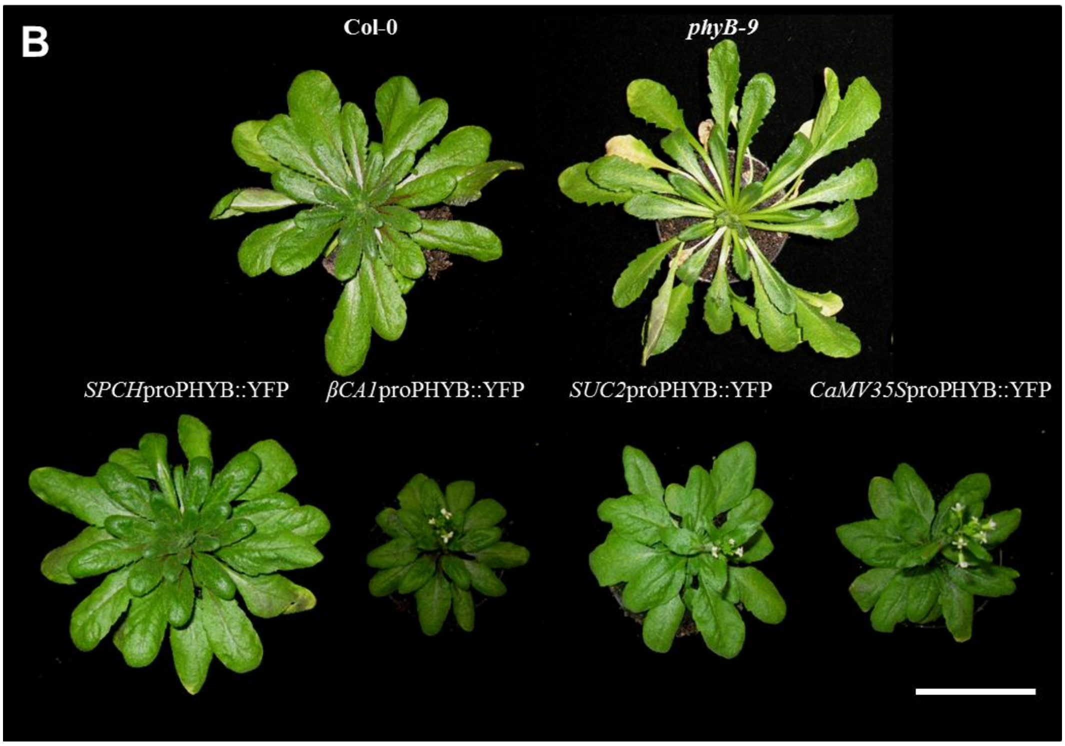

Figure S1, related to Figure 1. Tissue specific expression of *PHYB::YFP*.

A) Schematic detailing the expression patterns of the different tissue-specific promoters in leaf tissue as described in this study. Red indicates cells expressing PHYB::YFP

B) Rosette phenotype of mature plants (scale bar = 5cm).

Figure S2, related to Figure 2

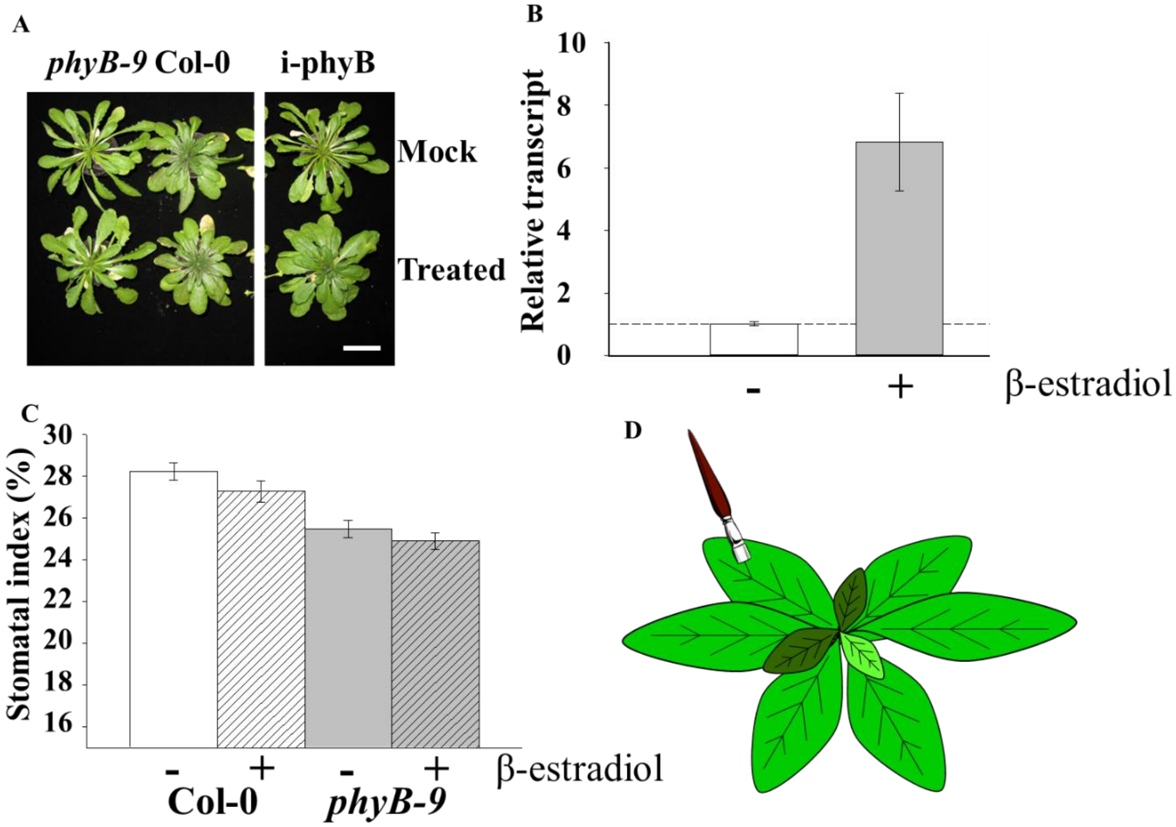

Figure S2, related to Figure 2. Inducible phytochrome B.

A) Phenotype of mature plants grown as in Figure 2A (scale bar = 5cm).

B) Quantitative RT-PCR showing induction of *PHYB* expression following treatment with 5µM β-estradiol. 2 week old seedlings were sprayed with 5µM β-estradiol and RNA extracted from mock treated (-) and treated (+) plants 2 days post treatment. Expression is calculated relative to the untreated control and error bars show mean +/- SEM.

C) The SI of Col-0 and *phyB-9* plants mock treated (-) or treated with 5µM β-estradiol (+). Plants were grown at 250µmol m^{-2} s^{-1} and sprayed daily. Mean values are shown with error bars indicating mean +/- SEM. No significant difference was observed within genotypes.

D) For determining the systemic role of *PHYB*, 5µM β-estradiol was applied to mature leaves (1-12) with a fine paint brush but not to young leaves (dark and light green).

Figure S3, related to Figure 3

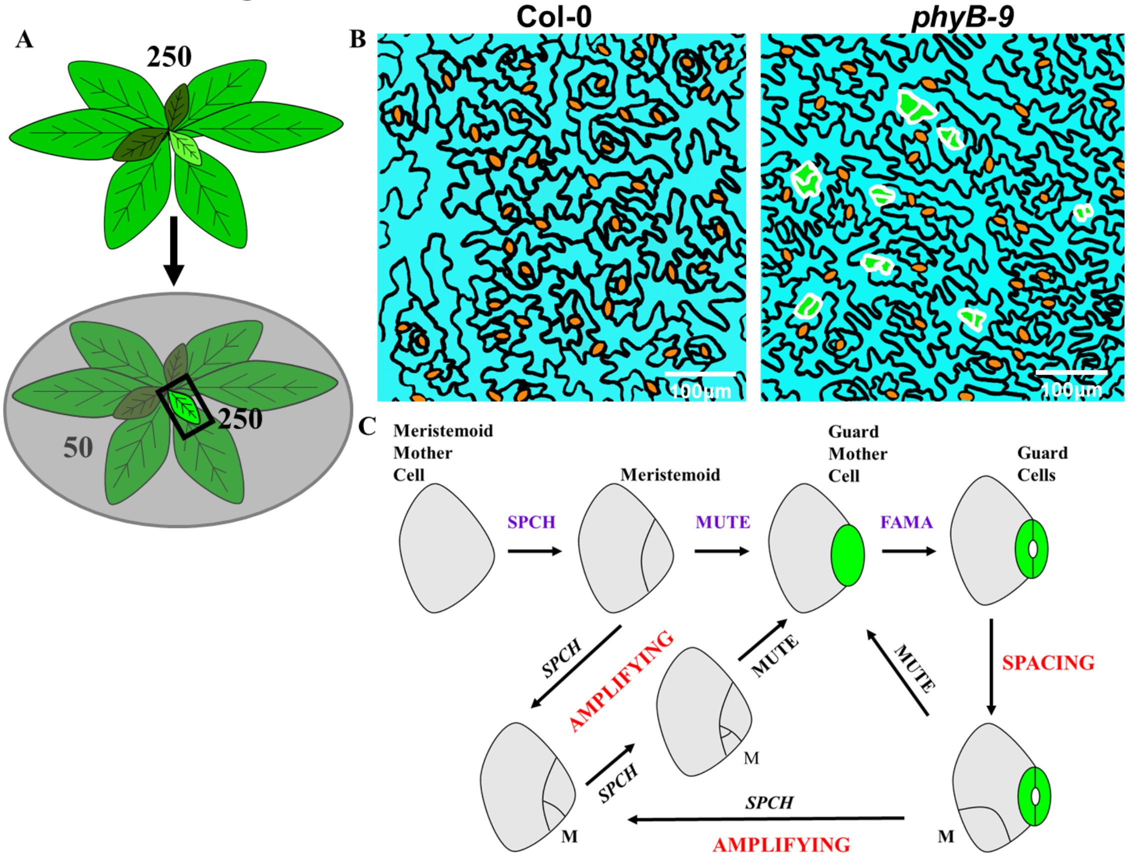

Figure S3, related to Figure 3. Scheme for shading and stomatal development

A) Plants were germinated at 250 µmol m^{-2} s^{-1} until leaf 14 primordia were visible. The existing leaves (greater than 5 mm; designated mature) were shaded to 50 µmol m^{-2} s^{-1} using neutral density filters whilst leaf 14 (light green) remained exposed to 250 µmol m^{-2} s^{-1}.

B) *phyB-9* mutants display aborted amplifying divisions. Epidermal tracings from mature leaves. Aborted divisions in the stomatal lineage are highlighted in white with cells filled in green; mature stomata are shown in orange (scale bar = 100µm).

C) A schematic indicating cell divisions within the stomatal lineage and the relevant roles of SPCH, MUTE and FAMA. Entry into the stomatal lineage occurs via an asymmetric division to generate a meristemoid cell, which requires SPCH. Differentiation into a Guard Mother Cell requires MUTE, whilst Guard Cell differentiation requires FAMA. Spacing and amplifying divisions result in further cells entering the stomatal lineage and generate satellite stomata.

Supplemental Experimental Procedures

Plant Material and Growth Conditions

Arabidopsis plants were all in the Col-0 ecotype. *phyB-9* is a strong mutant allele caused by a G-A transition leading to a premature STOP codon at amino acid 397 [8]. Phytochrome nomenclature is as detailed in [S1]. Therefore, phyB refers to the holoprotein, *PHYB* the wild-type gene, *phyB* the mutant and PHYB the apoprotein. Plants were grown in growth chambers (Snijder Microclima 1000E, Snijder Scientific, The Netherlands) from seed in 3:1 mix of compost-horticultural silver sand in short days (10 hr photoperiod, 70% RH, 22 °C) at a photon irradiance of 250 μmol m^{-2} s^{-1} unless otherwise stated.

Measurement of Stomatal Indices and Density

Impressions of the abaxial surface of mature rosette leaves, principal growth stage 5.10 [S2], were made with dental resin (President Jet Light Body, Coltene/Whaledent, Burgess Hill, UK). This was performed for leaves at comparable developmental stages. In the case of the tissue specific promoter analysis, two independent lines were analysed per construct. Clear nail varnish was applied to the set impression after removal from the leaf, and the varnish impressions were viewed on a Zeiss Axiovert 200M inverted microscope and imaged with Volocity software (Improvision Ltd, Coventry, UK). Stomatal and epidermal cell counts were taken from three areas per leaf with three leaves per plant from four separate plants and each experiment was performed in triplicate. For the density data, the mean was calculated from the total number of stomata or epidermal cells. The stomatal index was calculated for each area individually, and the mean was then calculated from these data. Stomatal index was calculated with the following formula: S.I. = [number of stomata/(number of other epidermal cells + number of stomata)] X 100. For statistical analysis, an unpaired t test was performed on the data following arcsine transformation, which was performed because stomatal index is a proportion and not a direct measurement.

Plasmid construction

PromoterPHYB::*YFP*

A full-length *PHYB* cDNA minus the TAG STOP codon was amplified from cDNA using phyBfor and phyBrev primers. This was then digested with *KpnI*, a site for which was added to the phyBrev primer. YFP was amplified from vector pGKGWY [S3] and was also digested with *KpnI*, a site for which was added to the YFPfor primer. The digested fragments were ligated together and then digested with *ApaI* (phyBfor primer) and *SbfI* (YFPrev primer). This product was then ligated into *ApaI-SbfI* digested pGKGWY to generate pGKGWY-*PHYB*. Promoters were amplified from genomic DNA (Col-0), digested with the relevant restriction endonculeases, and ligated into the *XbaI-ApaI* sites (*SPCH* 2060 bp; *SUC2* 2128 bp; *CaMV35S* 555 bp) or the *ApaI* site (*βCA1*1713 bp; At3g01500) of pGKGWY-*PHYB*.

Inducible PHYB

The Inducible *PHYB* line (i-*PHYB*) was generated using the β-estradiol inducible two-component vector system designed by Brand et al. [18]. The *PHYB* cDNA was amplified from Col-0 cDNA using the primers 221-phyBfor/rev and inserted into *AscI-PacI* digested pMDC221. A *CaMV35S* promoter was amplified from the binary vector pBI121 [S4] using the primers 150-35Sfor/rev and inserted into *AscI-PacI* digested pMDC150. Constructs were introduced into *phyB-9* mutants by the floral-dip method [S5] using the *Agrobacterium tumefaciens* C58C1 [S6]. Primary transformants were selected on growth medium supplemented with either 50 mg l^{-1} kanamycin (pMDC150-35S) or 20 mg l^{-1} hygromycin B (pMDC221-*PHYB*). Primary transformants displaying a *phyB-9* mutant phenotype (indicating a lack of non-specific transgene induction) were crossed and then selfed to generate a homozygous line containing both pMDC150-35S and pMDC221-phyB. Confirmation of

homozygosity for the *phyB-9* mutation was determined using the *phyB-9* specific primers *phyB-9*for/rev.

Details of primer sequences for vector construction can be found in the supplemental experimental procedures.

Confocal Microscopy

Confocal images were taken with a Leica TCS SP5 confocal microscope after counterstaining tissues with 10 µg ml^{-1} propidium iodide.

Gene expression analysis

For quantitative RT-PCR analysis (qPCR), RNA was extracted from 2 week old seedlings or leaves using the Qiagen RNeasy plant RNA extraction kit. 2µg of total RNA was treated with DNase according to the method of Sanyal et al. [S7], and then was reverse transcribed with Maxima reverse transcriptase (Fermentas). Transcript abundance of target genes was assayed using Maxima SYBR Green/ROX qPCR Master mix (Fermentas). The *ACTIN2* and *UBC21* genes were used as controls, as transcript levels remained constant under all treatments and relative expression levels were calculated using the ΔΔCt method [S8]. Expression was calculated relative to that of equivalent leaves from mock treated plants. Three biological repeats and three technical repeats were performed for each sample and used to calculate s.e.m. values. Reaction conditions were (1 x 95°C - 10 mins; 40 x 95°C - 15s/57°C – 20s/72°C – 30s). Details of primer sequences for gene expression analysis can be found in these supplemental experimental procedures.

Primers for quantitative RT-PCR

Primer	Forward (5'-3')<br>Reverse (5'-3')
ACTIN2	TCAGATGCCCAGAAGTGTGTT<br>CCGTACAGATCCTTCCTGATAT
UBC21	GAATGCTTGGAGTCCTGCTTG<br>CTCAGGATGAGCCATCAATGC
YFP	CTTCAAGGACGACGGCAACTAC<br>TTCAGCTCGATGCGGTTCAC
phyB	CCGTGACATTCCCGAAGAGAT<br>ATACTCAGCAGAAACTCAGCCA
SPCH	AACGGTGTCGCATAAGATCC<br>CAAGAGCCAAATCTTCAAGAGC
MUTE	AACGTCGAAAGACCCTAAACCG<br>TTAGCATGAGGGGAGTTACAGC
FAMA	GCTGCTAGGGTTTGACGCCATGA<br>GGAGTAGAGGACGGTTTGTTCC
EPF1	CCAACATCCTCCCATCCAAGTC<br>TGAGCAATCTGGCAACCTAGACC
STOMAGEN	GTTCAAGCCTCAAGACCTCG<br>CCTTCGACTGGAACTTGCTC

Primers for vector construction and genotyping

Primer	Forward (5'-3')<br>Reverse (5'-3')
phyB	TTAGGGCCCCAAACGGCATGGTTTCCGGAGTCG<br>TTAGGTACCATATGGCATCATCAGCATCATGTC
YFP	TTAGGTACCGTGGTGCTCGAGATGGTGAGC<br>CAGCGCATGCCTGCAGGTCGAC
SPCH	TTTCTAGAGTAACGGGCAACAGTGTTATAG<br>TTGGGCCCCGTGATTAGAGATATATCCT
SUC2	TTTCTAGAAAAATCTGGTTTCATATTAATTTCA<br>TTGGGCCCATTTGACAAACCAAGAAAGTAAGA
βCA1	TTGGGCCCGATCTGTAGGGTTGAGCAACTAGC<br>TTGGGCCCGCGAAGAGAAGCGGAGATCAGAG
CaMV35S	TTTCTAGAGAATTCCCATGGAGTCAAAAATTC<br>TTGGGCCCGGTACCGTGTCCTCTCCAAATGA
150-35S	GAGGCGCGCCTCAAATAGAGGACCTAACAG<br>AGTTAATTAATGTCCTCTCCAAATGA
221-phyB	TTGGCGCGCCCAAACGGCATGGTTTCCGGAGTCG<br>GGTTAATTAACCAACTGAAGTGTGACTAATATGGC
Wt-phyB	CTATGTGCTTGGTTGGTTCTAC<br>GATGGCAAACAACCAAACCC
phyB-9	GAAGCTCGATGAGGCTTTGA<br>CCATGATACTGGGACTCTGTG

Supplemental References

S1. Srivastava, L.M. (2002). Plant Growth and Development: Hormones and Environment (Elsevier Inc.).

S2. Boyes, D.C., Zayed, A.M., Ascenzi, R., McCaskill, A.J., Hoffman, N.E., Davis, K.R., and Gorlach, J. (2001). Growth stage-based phenotypic analysis of Arabidopsis: A model for high throughput functional genomics in plants. Plant Cell *13*, 1499–1510.

S3. Zhong, S., Lin, Z., Fray, R., Grierson, D. (2008). Improved plant transformation vectors for fluorescent protein tagging. Transgenic Res *17,* 985-989.

S4. Jefferson, R.A., Kavangh. T,A., Bevan, M.W. (1987). GUS fusions: β-glucuronidase as a sensitive and versatile gene fusion marker in higher plants. EMBO J *6*, 3901–3907.

S5. Clough, S.J., Bent, A.F. (1998). Floral dip: a simplified method for Agrobacterium-mediated transformation of Arabidopsis thaliana. Plant J *6*, 735–743.

S6. Dale, P.J., Marks, M.S., Brown, M.M., Woolston, C.J., Gunn, H.V., Mullineaux, P.M., Lewis, D.M., Kemp, J.M., Chen, D.F., Gilmour, D.M., Flavell, R.B. (1989). Agroinfection of wheat-inoculation of in vitro grown seedlings and embryos. Plant Sci *63*, 237–245.

S7. Sanyal, A., O'Driscoll, S.W., Bolander, M.A., Sarkar, G. (1997). An effective method of completely removing contaminating genomic DNA from an RNA sample to be used for PCR. Mol Biotechnol *8,* 135–137.

S8. Schmittgen, T.D., Livak, K.J. (2008). Analyzing real-time PCR data by the comparative C(T) method. Nat Protoc. *3*, 1101-1108.
